# Antiproliferative activity of platinum(II) and copper(II) complexes containing novel biquinoxaline ligands

**DOI:** 10.1093/mtomcs/mfae001

**Published:** 2024-01-05

**Authors:** Hager Sadek El-Beshti, Zuhal Gercek, Hakan Kayi, Yasemin Yildizhan, Yuksel Cetin, Zelal Adigüzel, Gamze Güngör, Şeniz Özalp-Yaman

**Affiliations:** Atilim University, Department of Chemical Engineering, Incek, Ankara, Türkiye; Zonguldak Bülent Ecevit University, Department of Chemistry, Incevez, Zonguldak, Türkiye; Ankara University, Department of Chemical Engineering, 06100, Tandoğan, Ankara, Türkiye; TUBITAK, Marmara Research Center, Life Sciences, Medical Biotechnology Unit, Gebze/Kocaeli, Türkiye; TUBITAK, Marmara Research Center, Life Sciences, Medical Biotechnology Unit, Gebze/Kocaeli, Türkiye; Koç University, School of Medicine, KUTTAM, Istanbul, Türkiye; TUBITAK, Marmara Research Center, Life Sciences, Medical Biotechnology Unit, Gebze/Kocaeli, Türkiye; Atilim University, Department of Chemical Engineering, Incek, Ankara, Türkiye

**Keywords:** Cu(II) and Pt(II) biquinoxalines, DNA/HSA binding, DNA cleavage, Apoptosis, Invasion/migration assay, ROS generation

## Abstract

Nowadays, cancer represents one of the major causes of death in humans worldwide, which renders the quest for new and improved antineoplastic agents to become an urgent issue in the field of biomedicine and human health. The present research focuses on the synthesis of 2,3,2ʹ,3ʹ-tetra(pyridin-2-yl)-6,6ʹ-biquinoxaline) and (2,3,2ʹ,3ʹ-tetra(thiophen-2-yl)-6,6ʹ-biquinoxaline) containing copper(II) and platinum(II) compounds as prodrug candidates. The binding interaction of these compounds with calf thymus DNA (CT-DNA) and human serum albumin were assessed with UV titration, thermal decomposition, viscometric, and fluorometric methods. The thermodynamical parameters and the temperature-dependent binding constant (Kʹ_b_) values point out to spontaneous interactions between the complexes and CT-DNA via the van der Waals interactions and/or hydrogen bonding, except Cu(ttbq)Cl_2_ for which electrostatic interaction was proposed. The antitumor activity of the complexes against several human glioblastomata, lung, breast, cervix, and prostate cell lines were investigated by examining cell viability, oxidative stress, apoptosis-terminal deoxynucleotidyl transferase-mediated dUTP nick end labeling, *in vitro* migration and invasion, *in vitro*-comet DNA damage, and plasmid DNA interaction assays. The U87 and HeLa cells were investigated as the cancer cells most sensitive to our complexes. The exerted cytotoxic effect of complexes was attributed to the formation of the reactive oxygen species *in vitro*. It is clearly demonstrated that Cu(ttbq)Cl_2_, Pt(ttbq)Cl_2_, and Pt(tpbq)Cl_2_ have the highest DNA degradation potential and anticancer effect among the tested complexes by leading apoptosis. The wound healing and invasion analysis results also supported the higher anticancer activity of these two compounds.

## Graphical Abstract

**Graphical Abstract:**
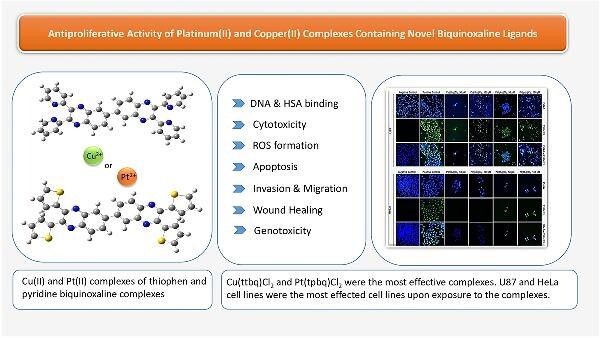
Antitumor activity of biqunoxaline complexes.

## Introduction

Chemotherapy, one of the alternatives that exists to fight cancer, relies on the discovery of chemicals that can kill rapidly dividing cells, thereby slowing and stopping the growth and spread of cancer.^1^ For decades, metal-based anticancer drugs have remained of great interest as a potential therapeutic agent against treatments^2^ or to detect disease.^3^ It goes without saying that a high level of success in this field owes itself to the application of cisplatin in hard tumor cases, which kills harmful cells and clears “the body of oncogenes.” Known as “cancer penicillin”^4^ because of its high potential to treat various forms of this disease, the use of cisplatin in chemotherapy has proven effective in the treatment of testicular cancer—among others—as it can rapidly alter the prognosis and cure up to 80% of cases within the early stages.^5^ However, despite this success, the use of cisplatin faces serious side effects that limit the dose administered to patients. In addition, this substance does not have sufficient efficacy in other cancer types, and tumors can often develop resistance to the drug over time.^6^ These limitations encourage experts to keep searching for metal-based drug alternatives to fight cancer by mimicking cisplatin which are now widely used in treatment and play a major role in medical tumors.^7–12^ Another strategy used to increase the efficacy of the antinoplastic agents is the use of chelating ligands. Combined chelated copper-based compounds, which are good candidates for this purpose, have been shown to possess improved antineoplastic properties compared to cisplatin when it comes to laboratory and clinical interventions.^13,14^ As a result, there are 2- and 3-Clip-Phen, which contain two phenanthroline ligands linked by a serinol bridge at the second or third position to better explain the coordination of the two phenanthroline units around the same copper ion. The oxidative nuclease processes of these compounds and molecules are far more active than phenanthroline. Clip-Phen compounds have been reported to have enhanced activity on DNA cleavage.^15^

In the light of the previously mentioned information, quinoxaline ligands were used in this study to obtain new metal-based anticancer compounds due to their increasing use nowadays.^16–18^ This effort could lead to numerous biological and therapeutic uses for quinoxaline or benzopyrazines, including their byproducts.^19,20^ As for quinoxaline cores, they have the ability to fight cancer, thus making them key ingredients in drugs for this purpose.^21^ For example, a new set of 2-alkylcarbonyl and 2-benzoyl-3-trifluoromethylquinoxaline-1,4-di-N-oxide derivatives was developed and evaluated for its antitumor properties against *in vitro* MCF7 (breast), NCIH 460 (lung), and SF-268 (CNS) models.^22^ Some previous research has already shed light on the effectiveness of pyridine platinum(II) compounds mimicked by cisplatin. These studies included peridyl-quinoxaline as a metal-based agent.^23^ Additionally, there is ample evidence to suggest that the interactions between platinum(II) compounds and quinoxaline ligands are key not only to the biological, but also the biochemical processes, thus paving the way for large-scale pharmaceutical production in recent years.^24^

**Fig. 1:**
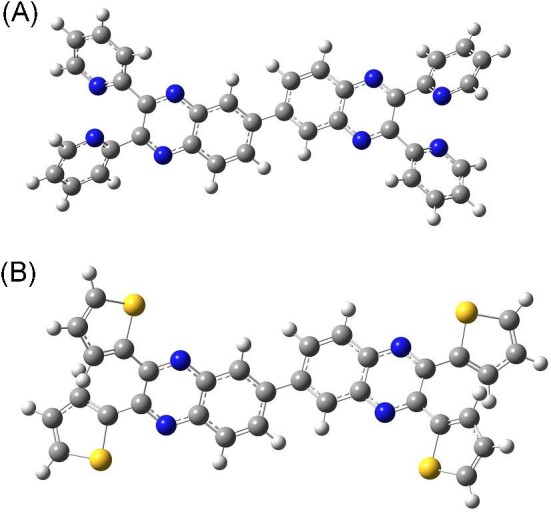
Quinoxalines used in the synthesis of the Pt(II) and Cu(II) complexes (A) 2,3,2ʹ,3ʹ-tetra(pyridin-2-yl)-6,6ʹ-biquinoxaline (tpbq) (B) 2,3,2ʹ,3ʹ-tetra(thiophen-2-yl)-6,6ʹ-biquinoxaline (ttbq).

Using the strategies summarized above, for the first time in this field as far as the literature is concerned, the present research combines two quinoxaline units (phenyl or thenyl quinoxalines) and, then, prepares their metal complexes to enhance the known anticancer effects of a single quinoxaline unit. For this purpose, the study reports on the synthesis, identification, and anticancer activity of 2,3,2ʹ,3ʹ-tetra(pyridin-2-yl)-6,6ʹ-biquinoxaline (tpbq or pyridyl quinoxaline)^25^ and 2,3,2ʹ,3ʹ-tetra(thiophen-2-yl)-6,6ʹ-biquinoxaline (ttbq or thenyl quinoxaline) ligands and their new copper(II) and platinum(II)chloride complexes with the closed formula of M(tpbq)Cl_2_ and M(ttbq)Cl_2_ (M: Cu(II) or Pt(II)) (see Fig. 1). Here, we determined the actual binding mode of the action between the compounds and DNA as well as blood transfer protein [human serum albumin (HSA)] using thermal denaturation and spectroscopic (electronic, fluorescence) and viscometric measurements. In addition, the bioefficacy of these complexes were investigated by testing the cytotoxicity, oxidative stress, apoptosis, invasion and migration inhibition capacity, and gonotoxicity potential using *in vitro* systems.

## Experimental

### Synthesis of ligands

The 2,3,2ʹ,3ʹ-tetra(pyridin-2-yl)-6,6ʹ-biquinoxalinyl (tpbq) and 2,3,2ʹ,3ʹ-tetra(thiophen-2-yl)-6,6ʹ-biquinoxaline (ttbq) were synthesized according to the following procedure:

A mixture of 2 mmol 2-dipyridyl ethandione, or mixture of 2 mmol of 2ʹ-dithienyl ethandione, with 1 mmol 3,3ʹ-diaminobenzidine and two drops of glacial acetic acid were refluxed in 25 ml ethanol for 5 h. After the reflux period, the reaction mixture was poured into 75 ml ice water and neutralized with a 30% NH_4_OH solution. The resultant precipitate was filtered and recrystallized in methanol. Green crystals of tpbq were obtained with 87% yield, and yellow crystals of ttbq were obtained with 65% yield.

tpbq: Anal. calc. for C_36_H_22_N_8_ (%): C, 76.240; H, 3.883; N, 19.766. Found C, 74.80; H, 4.03; N, 19.20. IR (ν/cm^−^^1^): 3100 w, 3054 w, 3003 w, (Ar-H), 1609 m (C=N), 1585 s (C=C), 1148 m (C-N), 1469 s (ph), 1277 m (C-H),1073 m, 995 m (C-H out of plane ring), 790 s, 748 vs. (C-C out of plane), 1347 s (C-H in plane), 1433 s (C-CH in plane). ^1^H NMR (600 MHz, CDCl_3_) δ 8.65 (2H, s, 5- and 5′-H), 8.41 (4H, d, J = 6 Hz, 4 × 6ʺ-H), 8.38 (2H, d, J = 12 Hz, 8- and 8′-H), 8.30 (2H, d, J = 12 Hz, 7- and 7′-H), 8.08 (4H, t, J = 12 Hz, 4 × 4ʺ-H), 7.91 (4H, 2xd, J = 12 Hz, 4 × 3ʺ-H), 7.31 (4H, m, 4 × 5ʺ-H).^13^C-NMR (CDCl_3_, 400 MHz, δ, ppm): 123.43, 124.67, 127.77, 130.25, 137.45, 141. 41, 141.74, 147.88, 156.63 (Fig. B1). ESI–MS (m/z, DCM): 566.63 (calc. 566.82) [M]^+^, 567.628 (calc. 567.20) [(tpbq)+H^+^] (Supplementary Fig. S1, S7 and S9).

ttbq: Anal. calc. for C_32_H_18_S_4_N_4_ (%): C, 65.442; H, 3.068; N, 9.544; S, 21.814. Found C, 65.10; H, 2.96; N, 9.09; S, 18.60. IR (ν/cm^−^^1^): 3095 w, 3062 w, 2921 w (Ar-H), 1610 m (C=N), 1519 m (C=C), 1184 w (C-N), 1477 s (ph), 1299 s (C-H),1079 w, 1058 w, 1041 w (C-H out of plane ring), 748 s, 698 vs. (C-C out of plane), 1358 s (C-H in plane), 1431 s (C-CH in plane), 833 m (C-S-C). ^1^H NMR (600 MHz, CDCl_3_) δ 8.55 (2H, s, 5- and 5′-H), 8.28 (2H, d, J = 6 Hz, 8- and 8′-H), 8.21 (2H, d, J = 6 Hz, 7- and 7′-H), 7.56 (4H, d, J = 6 Hz, 4 × 5ʺ-H), 7.36 (4H, s, 4 × 3ʺ-H), 7.08 (4H, s, 4 × 4ʺ-H).^13^C-NMR (CDCl_3_, 400 MHz, δ, ppm): 123.50, 126.92, 127.31, 129.25, 129.70, 139.30, 142.25, 144.81 (Fig. B2). ESI–MS (m/z, DCM): 586.78 (calc. 587.05) [M]^+^, 587.78 (calc. 588.05) [(ttbq)+H^+^] (Supplementary Figs. S2, S8, and S10).

### Synthesis of complexes

#### Copper complexes

In order to synthesize the 2,3,2ʹ,3ʹ-tetra(pyridin-2-yl)-6,6ʹ-biquinoxalinyl (Cu(tpbq)Cl_2_) and 2,3,2ʹ,3ʹ-tetra(thiophen-2-yl)-6,6ʹ-biquinoxaline (Cu(ttbq)Cl_2_) complexes of Cu(II), 0.13 g (0.23 mmol) tpbq or0.03 g (0.05 mmol) ttbq were fully dissolved in about 10 ml ethanol and reacted dropwise with an aqueous copper chloride (CuCl_2_) solution. Then, the solution was refluxed at 45–50°C for 24 h. The solid formed during this stage was collected by filtration and dried under vacuum. The yield percentage was 13.71% for Cu(tpbq)Cl_2_, and 26.08% for Cu(ttbq)Cl_2_.

Cu(tpbq)Cl_2_: Anal. calc. for C_36_H_22_N_8_Cl_2_Cu (%): C, 61.676; H, 3.163; N, 15.983. Found C, 61.466; H, 3.125; N, 15.599. IR (ν/cm^−^^1^): 3054 w, 3005 w, 2924 w, (Ar-H), 1706 m (C=N), 1577 s (C=C), 1149 m (C-N), 1472 s (ph), 1290 m (C-H),1072 m, 993 m (C-H out of plane ring), 789 s, 742 vs. (C-C out of plane), 1386 s (C-H in plane), 1430 s (C-CH in plane), 3366 w (HOH). Dispersive Raman (785 cm^−1^): 246 m, 353 m (M-Cl), 394 m, 460 m (M-N), 186 m (Cl-M-Cl), 220 w (Cl-M-N), 114 m (N-M-N). ^1^H-NMR [dimethyl sulfoxide (DMSO)-d_6_, 400 MHz, δ, ppm): δ :7.61 (d, 2H, H-5, 5′), 7.80 (m, 4H, H-3, 3′, H-4,4′), 8.24(d, 2H, H6,6′), 8. 41 (m, 2HC6H3),8.60 (s, HC6H3). UV-vis [(dimethylformamide (DMF)] λ_max_/nm (ɛ_max_/M^−1^cm^−1^):285 (2310), 373 (13490). ESI–MS (m/z, EtOH): 701.063 (calc. 701.064) [M]^+^, 382.32 (calc. 382.0) [Cu(tpbq)Cl]^+^, 699.41 (calc. 700.07) [Cu(tpbq)Cl]^+^-2H, 630.12(calc. 631.16) [Cu(tpbq)]^+2^, 417.223 (calc. 418.76) [CuCl_2_]^+^, 567.20 (calc. 566.63) [(tpbq)_2_ + 1], 284.32 (calc. 283.31) [tpbq + 1] (Supplementary Figs. S3, S11, S15, and S19).

Cu(ttbq)Cl_2_: Anal. calc. for C_32_H_18_S_4_N_4_Cl_2_Cu (%): C, 53.291; H, 2.516; N, 7.768; S, 17.783. Found C, 53.655; H, 2669; N, 7.548; S, 17.348. IR (ν/cm^−^^1^): 3053 w, 2922 w (Ar-H), 1607 m (C=N), 1519 m (C=C), 1179 w (C-N), 1473 s (ph), 1296 s (C-H),1045 w, 978 w, 934 w (C-H out of plane ring), 744 s, 696 vs. (C-C out of plane), 1368 s (C-H in plane), 1423 s (C-CH in plane), 835 m (C-S-C), 3462 w (HOH). Dispersive Raman (785 cm^−^^1^): 239 m, 280 m (M-Cl), 381 m, 417 m (M-N), 146 m (Cl-M-Cl), 206 w (Cl-M-N), 120 m (N-M-N). ^1^H-NMR (DMSO-d_6_, 400 MHz, δ, ppm): 7.21 (d, 2H, H-3,3′), δ: 7.30 (t,2H, H-4,4′), 8.00 (dd, 2H, H-5,5′), 8.48 (dd, 2H, C6H3),8.60 (s, HC6H3). UV-vis (DMF) λ_max_/nm (ɛ_max_/M^−1^cm^−1^): 287 (55990), 415 (58830). ESI–MS (m/z, Acetonitrile): 721.51 (calc. 721.22) [M]^+^, 723.51 (calc. 723.22) [Cu(ttbq)Cl_2_]+H^+^, 685.44 (calc. 685.78) [Cu(ttbq)Cl]^+^, 649.18 (calc. 650.33) [Cu(ttbq)]^+2^, 743.52 (calc. 744.23) [Cu(ttbq)Cl_2_]^+2^ + Na^+^,663.46 (calc. 662.78) [Cu(ttbq)]^+2^-(Cl + Na), 587.05 (calc. 586.78) [(ttbq)_2_](Supplementary Figs. S4, S12, S16, and S19).

#### Platinum complexes

The synthesis of the platinum(II) complexes of tpbq and ttbq followed two steps. In the first step, 163.7 mg (0.964 mmol) AgNO_3_ was directly added into a 5 ml aqueous solution of 100 mg (0.241 mmol), K_2_PtCl_4_ in darkness and left overnight. In the second step, after removing the precipitated AgCl, 5 ml DMF solution of 0.136 g (0.482 mmol) (tpbq) or 0.1413 g (0.482 mmol) (ttbq) was added dropwise to the Pt- solution while stirring. Next, the solution was refluxed at 40°C for 24 h, and the precipitate of the Pt-complexes was collected under vacuum and dried at room temperature. The percentage yields obtained for the Pt(tpbq)Cl_2_ was 16.45% and 3.19% for Pt(ttbq)Cl_2._

Pt(tpbq)Cl_2_: Anal. calc. for C_36_H_22_N_8_Cl_2_Pt (%): C, 51.932; H, 2.663; N, 13.458. Found C, 52.890; H, 2.588; N, 13.335. IR (ν/cm^−^^1^): 3200 w, 3055 w, 2915 w, (Ar-H), 1707 m (C=N), 1580 s (C=C), 1152 w (C-N), 1472 s (ph), 1293 m (C-H),1076 w, 1042 w (C-H out of plane ring), 789 s, 744 vs. (C-C out of plane), 1380 s (C-H in plane), 1426 s (C-CH in plane), 3365 w (HOH). Dispersive Raman (785 nm): 340 m (M-Cl), 404 m (M-N), 113 m (Cl-M-Cl), 213 w (Cl-M-N), 240 m (N-M-N). ^1^H-NMR (DMSO-d_6_, 400 MHz, δ, ppm): δ :7.59 (d, 2H, H-5, 5′), 7.83 (m, 4H, H-3, 3′, H-4,4′), 8.27 (d, 2H, H6,6′), 8. 50 (m, 2HC_6_H_3_), 8.68 (s, HC_6_H_3_). UV-vis (DMF) λ_max_/nm (ɛ_max_/M^−1^cm^−1^): 287 (67930), 373 (43540). ESI–MS (m/z, EtOH): 832.37 (calc. 832.60) [M]^+^, 799.01 (calc. 797.65) [Pt(tpbq)Cl]^+^, 762.04 (calc. 762.20) [Pt(tpbq)]^+2^, 764.04 (calc. 764.20) [Pt(tpbq)]^+2^ + 2H, 587.51 (calc. 587.63) [(tpbq) +2Cl]^+^-2H, 567.20 (calc. 566.63) [(tpbq)], 284.10 (calc. 283.31) [tpbq] (Supplementary Figs. S5, S13, S17, and S19).

Pt(ttbq)Cl_2_: Anal. calc. for C_32_H_18_S_4_N_4_Cl_2_Pt (%): C, 45.070; H, 2.128; N, 6.570; S, 15.039. Found C, 45.264; H, 2.213; N, 6.604; S, 15.408. IR (ν/cm^−^^1^): 3054 w, 2924 w, (Ar-H), 1606 w (C=N), 15198 w (C=C), 1178 w (C-N), 1473 m (ph), 1295 m (C-H),1048 w, 978 w, 933 m (C-H out of plane ring), 744 s, 695 vs. (C-C out of plane), 1368 m (C-H in plane), 1422 m (C-CH in plane), 835 m (C-S-C), 3358 w (HOH). Dispersive Raman (785 cm^−^^1^): 388 m (M-Cl), 528 m (M-N), 118 m (Cl-M-Cl), 200 w (Cl-M-N), 250 m (N-M-N). ^1^H-NMR (DMSO-d_6_, 400 MHz, δ, ppm): 7.15 (d, 2H, H-3,3′), δ: 7.36 (t, 2H, H-4,4′),8.15(dd, 2H, H-5,5′), 8.50 (dd, 2H, C6H3),8.60 (s, HC6H3). UV-vis (DMF) λ_max_/nm (ɛ_max_/M^−1^ cm ^−1^): 284 (49140), 414 (48890). ESI–MS (m/z, CH_2_Cl_2_): 852.95 (calc. 852.77) [M]^+^, 815.51 (calc.815.32) [Pt(ttbq)Cl_2_] -[Cl + 2H], 910.39 (calc. 910.75) [Pt(ttbq)Cl_2_] +[Na + K]-5H, 851.39 (calc. 851.77) [Pt(ttbq)Cl_2_] -H, 891,54 (calc. 892.76) [Pt(ttbq)Cl_2_] +K, 587.05 (calc. 586.78) [(ttbq)](Supplementary Figs. S6, S14, S18, and S19).

### DFT calculations

Density functional theory calculations were performed by utilizing the hybrid functional B3LYP^26–29^ and lanl2dz basis set for the computational analysis of the metal ligand complexes as described in our previous study.^30,31^ Grimme's empirical dispersion corrections with Becke–Johnson damping were performed for all DFT calculations by using the Gaussian 09 Rev. D.01 software package.^32^

### Binding studies

The DNA and HSA binding studies were carried out using the methods described in our previous study.^31^ The details of the synthesis and the identification of the compounds can be found elsewhere.^33–36^

The change in the electronic absorption spectrum of the complexes in the presence and the absence of CT-DNA was followed using an HP Agilent8453 Spectrophotometer. During the measurements, the concentration of the compounds was kept constant [Cu(tpbq)Cl_2_ = 3.0 × 10^−3^ M; Cu(ttbq)Cl_2_ = 0.50 × 10^−3^ M; Pt(tpbq)Cl_2_ = 3.0 × 10^−3^ M; Pt(ttbq)Cl_2_ = 3.0 × 10^−3^ M)], while the CT-DNA concentration was changed depending on the R (R=[DNA]/[complex]) value of 0 to 10. The stock solutions of all the complexes were prepared in DMF and, then, diluted to the desired concentrations using a 5 mM Tris HCl-50 mM NaOH buffer (1:1) at pH = 7.11 prior to the UV titrations, where the percentage ratio of DMF in the final solutions did not exceed 0.5%. The optimal incubation time was determined as 30 min for Cu(ttbq)Cl_2_, 60 min for Pt(tpbq)Cl_2_, and 45 min for Cu(tpbq)Cl_2_ and Pt(ttbq)Cl_2_ at 37°C spectroscopically. The intrinsic binding constant (K_b_) and the thermodynamic parameters of our compounds were calculated according to the change in the intensity of the electronic absorption band observed at 375 nm for Cu(tpbq)Cl_2_, 412 nm for Cu(ttbq)Cl_2_, 376 nm for (Pt(tpbq)Cl_2_, and 421 nm for (Pt(ttbq)Cl_2_ using the methods available in literature.^1–31,33,34^ Similar experiments were carried out to show the strength of binding of our complexes (K_b_) to HSA and to investigate the related thermodynamic parameters in the same buffer solution as well.^31^ In these studies, 2.12 × 10^−5^ M HSA titrated with the complexes (0 and 2.12 × 10^−6^ M) and the spectral changes in the electronic absorption spectrum were monitored at 280 nm. The optimal incubation time for HSA binding titrations was determined as 30 min for Cu(ttbq)Cl_2_ and Pt(ttbq)Cl_2_, and 60 min for Cu(tpbq) Cl_2_ and Pt(tpbq)Cl_2_ spectroscopically.^37^

The thermal decomposition of 60 μM CT-DNA in the presence of the compounds was monitored spectroscopically at 260 nm and between 30°C and 90°C with 10 to 160 μM of the complexes in a HAAKE temperature-monitored circular bath. Similarly, the change in the viscosity of 60 μM CT-DNA was studied with the help of an AND SV-10 VIBRO Viscometer by varying the concentration of the compounds between 10 and 70 μM at room temperature.^35^

The HSA thermal decomposition was followed at 280 nm upon increasing the temperature from 30°C to 130°C at a 1°C min^−1^ rate from the spectral change. In order to better examine the processes involving the interaction of metal complexes and protein structure, viscometric titration was carried out in the same manner as for the nucleic acid.

In order to obtain the linear Stern–Volmer quenching constant (K_sv_), a Thermo-Scientific Lumina Fluorescence Spectrometer was used to conduct the flourometric measurements of 10 μM ethidium bromide (EtBr)-pretreated CT-DNA solutions in the presence of 0 to 160 μM compounds.^31,36^ The excitation wavelength was adjusted at 478 nm, and emissions ware observed at about 610 nm.

Tests involving fluorescence titration were conducted by maintaining the concentration of 1 μM HSA and changing the concentrations of the complexes between 0 and 50 μM in 5 mM Tris HCl buffer. The fluorescence spectral measurements were made at 200 to 500 nm after exciting the solution at 280 nm.

### *In vitro* bioefficacy studies

All the experiments conducted in this section were performed precisely as explained in our previous study.^31^ The cytotoxicity, oxidative stress, apoptotic effects, and antitumor activities of the synthesized complexes were investigated by using several human glioblastomata, prostate, breast, and lung cell lines and non-cancer cell lines used as control.

Cell culture growth conditions: Selected cancer cell lines from different origins, including human glioblastoma A172 (CRL-1620), LN229 (CRL-2611), U87 (HTB-14) cell lines, human cervix HeLa (ATCC, CCL-2), human breast MDA -231 (HTB-26), human prostate PC-3 (ATCC, CRL-1435), human lung A-549 (CCL-185), and non-cancer Chinese hamster ovary CHOK1 cell line (CCL61) (which was used as control) were purchased from American Type Culture Collection (ATCC, USA). These cultures were grown in Dulbecco's modified Eagle's medium-F12 (DMEM/F12, #D0547, Sigma-Aldrich) supplemented with 5% fetal bovine serum (#10500, Gibco) and 1% antibiotic-antimycotic (#15240, Gibco), and incubated at 37°C and 5% CO_2_. They were harvested and used for the following assays after reaching 70–80% confluency.

#### MTT cell viability assay

The used cell lines (A172, LN229, U87, HeLa, MDA-231, PC-3, A549, and CHO-K1) were harvested and seeded (6 × 10^3^ cells/well) into the 96-well plates and incubated at 37°C and 5% CO_2_ for 24 h. After that, the synthesized Cu(II) and Pt(II) complexes, at a concentration range of 6.25 to 100 μM in DMEM-F12, were added to the cell monolayers and incubated at 37°C and 5% CO_2_ for 24, 48, and 72 h. The dose–response relationships of the synthesized complexes were determined by using an MTT colorimetric agent (3-[4,5-dimethylthiazol-2-yl]-2,5-diphenyltetrazolium bromide, #M5655, Sigma-Aldrich), which is reduced by the mitochondrial enzymes of the metabolically active live cells and converted into a purple formazan product. The MTT stock solution was later dissolved in a phosphate buffer solution (PBS, 5 mg/ml, w/v) and, then, 5 μg/ml of MTT in DMEM was added into each well and incubated at 37°C and 5% CO_2_ for 4 h. DMSO (#D 8418, Sigma-Aldrich), as a dissolving solution for formazan crystals, was added into each well and placed in a shaker for 2 h. The absorbance at a test wavelength of 570 nm and a reference wavelength of 630 nm was measured on a microplate reader.

#### Oxidative stress testing—DCFDA assay

The reactive oxygen species (ROS) formation on the selected cell lines upon exposure to the synthesized Cu(II) and Pt(II) complexes was investigated using the oxidation-sensitive dye, 2ʹ,7ʹ-dichlorofluorescein diacetate (DCFDA, #D6883 Sigma-Aldrich). The cell lines (A172, LN229, U87, HeLa, MDA-231, PC-3, A549, and CHO-K1) were seeded (6 × 10^3^ cells/well) into the 96-well plates and incubated at 37°C and 5% CO_2_ for 24 h. After that, the Cu(II) and Pt(II) complexes, at a concentration range of 6.25 to 100 μM, were added to the cell monolayers. Hydrogen peroxide (H_2_O_2_), at concentrations from 50 to 500 μM, was utilized as a positive control. The following day, 5 μM of DCFDA in PBS was added to the cell monolayers and, then, the plate was incubated for 30–45 min. Consequently, the fluorescence intensity was measured with 485 nm excitation and 535 nm emission wavelengths using a microplate reader. The fluorescence values of each treatment were normalized to the negative control, including only growth medium without any treatment. The assay was repeated in triplicate.

#### Tunnel assay

The sensitive U87 and HeLa cell lines, selected according to the results of the IC_50_ values of the examined Cu(II) and Pt(II) complexes, were seeded at a seeding cell number of 5 × 10^4^ cells on the 12 mm round cover slips placed in the 24-well plate and incubated at 37°C and 5% CO_2_ for 24 h. The following day, the Cu(dpq)Cl_2_, Cu(dtq)Cl_2_, Pt(dpq)Cl_2_ and Pt(dtq)Cl_2_ complexes were freshly prepared and added at a range of 50 and 100 μM to the cell monolayers. After a 24 h incubation time, the cell monolayers were fixed with 4% paraformaldehyde for 15 min at room temperature and, then, the terminal deoxynucleotidyl transferase (TdT)-mediated dUTP nick end labeling (TUNEL) method was applied to detect the DNA damage associated with apoptosis. A commercial assay kit, In Situ Cell Death Detection Kit (Roche, #11767291910), was used for this purpose in accordance to the manufacturer's instructions. The experiment was repeated twice with the tested cell lines, and images were recorded with using a Leica DMI 6000 fluorescence microscope with 40× magnification.

#### Matrigel invasion analysis

The analysis was completed with U87 and HeLa cell lines to assess the metastatic potential of tumor cells using transwell inserts with an 8 μm pore size polycarbonate membrane (Corning Costar, Cambridge, MA, USA) coated with 1/5 of Matrigel (Beckton Dickinson, Bedford, MA, USA) and air-dried at room temperature. After seeding the U87 and HeLa cell lines (3 × 10^5^cells/insert), the complex concentrations were selected as 6.25 or 12.5 μM and incubated at 37°C in a humidified incubator with 5% CO_2_ for 24 h. The cell monolayers in the transwell inserts were fixed with ice-cold methanol and stained with 0.05% crystal violet for the analysis. The invasion of the cells to the basolateral side of the inserts was investigated using a light microscope with 40× magnification.

#### *In vitro* scratch wound healing

In vitro scratch wound healing experiments were carried out with HeLa cell lines (3 × 10^4^ cells/insert), which were seeded into 6-well plates. When they reached 80–90% confluence, scratches were created using AutoScratch (BioTek) in a straight line. The same Cu(II) or Pt(II) complex concentrations (6.25 or 12.5 μM) were used and incubated in the presence of 5% CO_2_ at 37°C in a humidified incubator for 24, 48, and 72 h. Images were obtained at the beginning of the exposure (time 0), and repeated at the end of 24, 48, and 72 h incubation periods.

#### *In vitro* comet assay

Commet assay was performed with U87 and HeLa cell lines (4 × 10^4^ cells/well) to detect DNA damage in the individual eukaryotic cells, where 25 or 50 μM Cu(II) or Pt(II) complexes were added to the cells and incubated for 24 h. Subsequently, the comet assay was performed as described before.^31^ The DNA damage was evaluated by calculating the DNA tail percentage using the following equation: DNA tail % = 100× tailing DNA density/cell DNA density. The assay was repeated twice, and 50 individual cells were involved for each treatment.

#### DNA cleavage activities

The DNA helix cleavage effect of the synthesized Cu(II) or Pt(II) complexes was evaluated by determining their ability to convert the plasmid DNA in the supercoiled form (SC) to its nick circular form (NC) and linear form (LF). pBI-CMV1 plasmid (3.1 kb) was grown in *Escherichia coli* and, then, purified using a Machery Nagel DNA isolation kit. The Cu(II) or Pt(II) complexes, at concentrations of 100 or 200 μM in the water, were incubated with 100 ng of plasmid DNA in double-distilled water (ddH_2_O) for 16 h at ambient temperature in a total volume of 20 μl. After that, the samples under investigation were electrophoresed on 1% agarose gels by means of a tris-acetate-EDTA (TAE) buffer at 100 V for 1 h. The gel was stained using EtBr, and images of the bands were captured by a ChemiDoc imaging system (BioRad).

Statistical analyses were performed by ANOVA and Tukey's post hoc test at a *P* < 0.05 significance level.

All complexes and ligands are stable in DMF and buffer solutions for 24, 48, and 72 h and longer. The stability of the complexes was checked using electronic absorption spectrum before the experiments.

## Results and discussion

### Synthesis and characterization

The ligands used in the present study, tpbq and ttbq, contain two quinoxaline groups attached from their 6-6′ position, as depicted in Fig. 1. The ligands and their copper(II) and platinum(II) complexes were stable at room temperature, sparingly soluble in ethanol and methanol but very soluble in DMF. The elemental analysis and mass spectrum results support the composition of the compounds. Furthermore, the change in the position of the proton signals of tpbq and ttbq in DMSO-d_6_ in the ^1^H-NMR spectrum of the metal complexes with metal ions was a good indication of metal ion-ligand coordination. In those spectra, the proton signals of tpbq, observed between δ: 7.29 and 8.65 ppm, deviate to δ: 7.42 and 8.34; likewise, the proton signals of ttbq at around δ: 7.24 and 8.54 ppm shift toward δ: 7.15 and 8.60 ppm upon metal coordination.

The electronic absorption spectrum of the metal complexes had two main absorption bands in DMF (Supplementary Fig. S19). The band, which appeared at about 284–287 nm, was attributed to the π→π* charge transfer transitions.^38^ Furthermore, the band observed at around 373 nm for the Cu(tpbq)Cl_2_ and Pt(tpbq)Cl_2_, and at 415 nm for the Cu(ttbq)Cl_2_ and Pt(ttbq)Cl_2_ complexes were assigned to the n→π* transitions.^39,40^

The aromatic C-H vibration absorption peaks appeared in the FTIR spectrum of tpbq at approximately 3100, 3054, and 3003 cm^−1^. Following the coordination of Cu(II) and Pt(II) to tpbq, those absorption bands shifted to 3200, 3055, and 2924 cm^−1^. Similar to this, upon metal coordination, the aromatic C=N vibration at 1609 cm^−1^, the ν_(C=C)_ (C=C) stretching vibration at 1585 cm^−1^, and the ν_(ph)_ vibration at 1468 cm^−1^ for tpbq appeared at approximately 1606, 1580, and 1472 cm^−1^, respectively.

The aromatic (C-H) vibrations in the FTIR spectrum of ttbq were identified as the source of the absorption peaks, which appeared at approximately 3095, 3062, and 2921 cm^−1^. Here, the ν_(C-24)_ vibrations shifted to 3053, 2924, and 2856 cm^−1^ due to metal-ttbq coordination. Similarly, for Cu(ttbq)Cl_2_ and Pt(ttbq)Cl_2_, the aromatic ν_(C=N)_ vibration frequency at 1600 cm^−1^ was observed at 1607 cm^−1^, and the ν_(ph)_ vibration frequency of ttbq, observed at roughly 1477 cm^−1^, appeared at 1473 cm^−1^. The absorption peak at around 833 cm^−1^ was ascribed to the ν_(C-S-C ring)_ for ttbq and was seen almost at the same frequency (835 cm^−1^) for Cu(ttbq)Cl_2_ and Pt(ttbq)Cl_2_, revealing no bond formation between metal and sulfur atom.

Additionally, the Raman spectrum of the complexes was taken in order to clarify the structure of our complexes by determining possible band vibrations between the metal and -N, -S or -Cl atoms. The vibrational modes, appearing at around 112 and 118 cm^−1^ in the Raman spectrum, were assigned to ν_(Cl-Pt-Cl)_ stretching for all Pt complexes. The Cl-Cu-Cl absorption, on the other hand, was observed at about 113–186 cm^−1^. These vibrations clearly indicated direct coordination of chlorine atom with the metal ions. Similarly, the ν_(N-Cl)_ band vibrations were obtained at around 340–381 cm^−1^ for both the tpbq and ttbq complexes. The Cl-M-N vibrations were detected at around 200–220 cm^−1^, while the absorption bands of the M-N vibrations for symmetric and asymmetric modes were seen at around 394 to 528 cm^−1^. Likewise, the N-M-N vibrational modes were obtained at around 114 cm^−1^ for the Cu(II) complexes and at about 240 cm^−1^ for the Pt(II) complexes. The data suggested that all the ligands coordinated with the metal ions through the N-atom of quinoxaline units, and that the chlorine atoms interacted with the metal centers directly in the inner shell of the complexes. It was very interesting to observe that no absorption band appeared at around 265 cm^−1^ for the Cu(ttbq)Cl_2_ and at about 280 cm^−1^ for the Pt(ttbq)Cl_2_ complexes. The absence of these bands could be attributed to Cu–S and Pt–S vibrations, respectively. These results clearly showed that the thenyl–quinoxaline ligands were not coordinated with the metal ions through S-atoms in our complexes. Similar results were obtained from the FTIR measurements.

### Computational studies

The geometry optimized structures of Cu(tpbq)Cl_2_ and Pt(tpbq)Cl_2_ display a deviation from the square planar structure around the metal center. This deviation becomes significant for Cu(tpbq)Cl_2_ with the N1-Cl4-N2-Cl3 dihedral angle of 49.3°. The deviation from planarity for Pt(tpbq)_2_Cl_2_ is rather slight by an N1-Cl4-N2-Cl3 dihedral of only 3.6° (Table 1).

**Table 1.** Selected geometrical parameters for the metal-ligand complexes (bond lengths are in Å, bond angles and dihedrals are given in °)

	Geometrical parameter M: metal center (Cu^2+^ or Pt^2+^)							
Complexes	M-N1	M-N2	M-Cl3	M-Cl4	N1-M-N2	Cl3-M-Cl4	N1-M-Cl4	N1-Cl4-N2-Cl3
Cu(tpbq)Cl_2_	2.10	2.02	2.28	2.28	78.9	101.8	144.5	49.3
Pt(tpbq)Cl_2_	2.05	2.03	2.40	2.40	79.6	87.7	172.6	3.6
	**M-N1**	**M-H2**	**M-Cl3**	**M-Cl4**	**N1-M-H2**	**Cl3-M-Cl4**	**N1-M-Cl4**	**N1-Cl4-H2-Cl3**
Cu(ttbq)Cl_2_	1.99	2.65	2.22	2.24	68.7	148.7	102.6	59.8
Pt(ttbq)Cl_2_	1.97	2.60	2.40	2.39	70.3	171.3	95.4	59.5

**Fig. 2:**
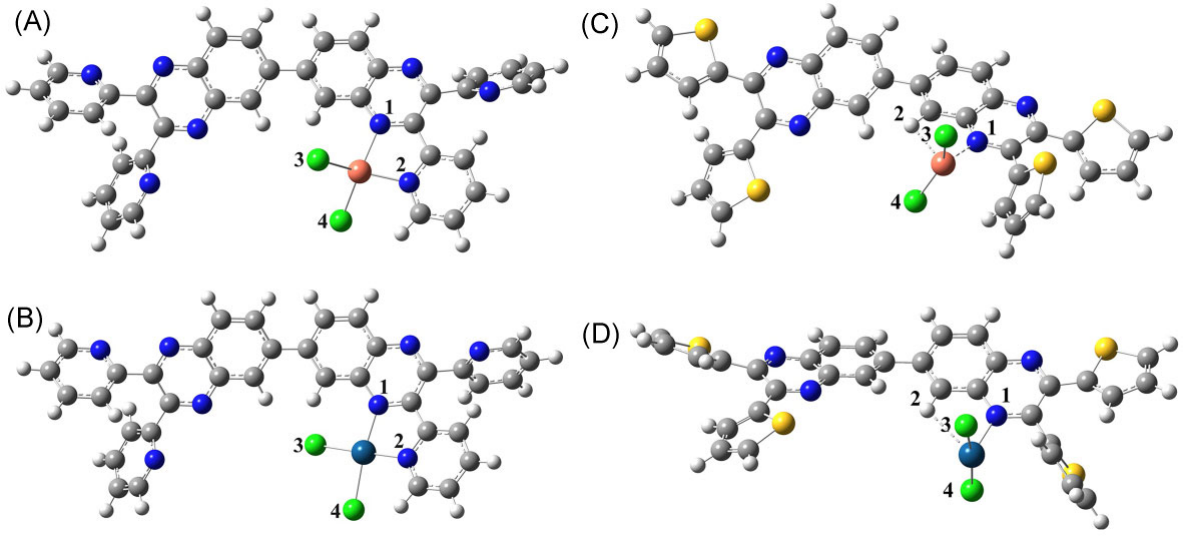
Optimized structures of (A) Cu(tpbq)Cl_2_, (B) Pt(tpbq)Cl_2_, (C) Cu(ttbq)Cl_2_, and (D) Pt(ttbq)Cl_2_ neutral complexes.

In the complexes mentioned earlier, Pt^2+^ interacts with the chlorine atoms through a longer distance compared to Cu^2+^. The Pt-N distances were calculated as 2.03 and 2.04 Å, while the Cu-N distances were found to be 2.10 and 2.02 Å. The optimized geometry calculations for Cu(ttbq)Cl_2_ and Pt(ttbq)Cl_2_, on the other hand, exhibited coordination between one of the H and N atom of the quinoxaline unit and the metal ions. The chlorine atoms filled the empty coordination sites as depicted in Fig. 2. The interaction of the metal ions with the H-atom led the deviation from planarity, which was also proved by a high N1-Cl4-N2-Cl3 dihedral of 59.8° and 59.5° for Cu(ttbq)Cl_2_ and Pt(ttbq)Cl_2_, respectively. For both complexes, the M^2+^-N and M^2+^-H distances were found to be almost equal, while longer metal ion-Cl distances were observed for the platinum complex than that of the copper complex (Table 1). It is evident from Fig. 2, no metal-sulfur atom coordination is seen in the optimized structure of Cu(ttbq)Cl_2_, and Pt(ttbq)Cl_2_. This was also parallel with the experimental findings.

### DNA-binding studies

**Fig. 3:**
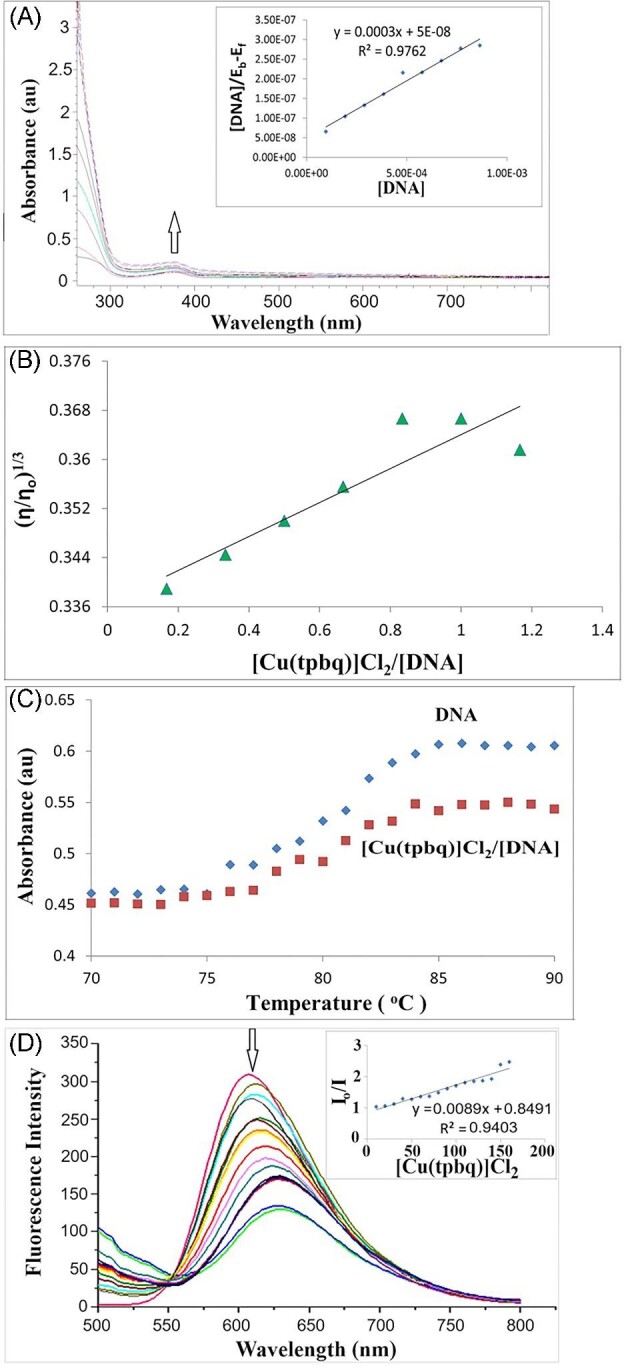
(A) Electronic absorption spectra of Cu(tpbq)Cl_2_ in the absence and in the presence of increasing amount of CT-DNA (0–1.25 × 10^−3^) (inset: the plot used for calculating K_b_). (B) Thermal denaturation plots obtained for Cu(tpbq)Cl_2_ and CT-DNA. (C) The changes in the relative viscosity of the CT-DNA in the presence of Cu(tpbq)Cl_2_. (D) The change in the Fluorescence spectrum of EtBr-bound CT-DNA in the presence of Cu(tpbq)Cl_2_ (10–160 μM) (inset: the plot used for calculating K_SV_).

Electronic absorption titration was performed to examine the type of the binding interaction of the complexes to the DNA—covalent or non-covalent. For this purpose, the electronic absorption spectra of the complexes were compared in the existence and the absence of CT-DNA. Cu(II) and Pt(II) complexes exhibited n→π* transitions^39,40^ at around 373 and 415 nm in the UV region. Upon increasing the concentration of CT-DNA, a hyperchromic change in the band intensity was seen, as illustrated in Fig. 3A and Supplementary Figs. S20a–S22a, while the peak position remained constant. Given that hyperchromism is often correlated with non-covalent binding,^41^ such a rise in the intensity of the electronic absorption band was most probably representative of electrostatic interactions occurring between the compound cations and the phosphate groups in the CT-DNA duplex^42^ after the chloride ions had been freed from the outer sphere.

The intrinsic binding constants (K_b_) for the present complexes were evaluated based on the spectral changes taking place in the compounds (Table 2; Fig. 3A, inset). The Pt(II) and Cu(II) complexes having tpbq show three times higher affinity toward CT-DNA compared to those with ttbq. This might be attributed to the different donation ability of pyridine- and thienyl-containing quinoxaline ligands to the metal ions. It was also observed that the Cu(II) tpbq and ttbq complexes maintained stronger interactions with the CT-DNA than those of Pt(II) (Table 2). These K_b_ values fall even lower compared to the value for commonly used intercalators,^43–46^ likely suggesting the presence of a rather weak non-covalent interaction between the compounds and CT-DNA.

**Table 2.** Thermodynamic data and fluorescence quenching constant values of the complexes upon binding to CT-DNA

Compounds	K_b_(M^−1^)	ΔG^o^(J)	ΔH^o^(J)	ΔS^o^(J/K)	K_SV_
Cu(tpbq)Cl_2_	6. 00 × 10^3^	−2.24 × 10^4^	−8.53 × 10^4^	−207.58	0.0105
Cu(ttbq)Cl_2_	2.00 × 10^3^	−1.96 × 10^4^	−1.15 × 10^4^	26.78	0.0137
Pt(tpbq)Cl_2_	2.22 × 10^3^	−1.99 × 10^4^	−3.82 × 10^4^	−59.89	0.0082
Pt(ttbq)Cl_2_	6.67 × 10^2^	−1.68 × 10^4^	−2.87 × 10^4^	−39.74	0.0143

K_b_: binding constant; ΔG^o^: standard Gibbs free energy change; ΔH^o^: standard enthalpy change; ΔS^o^: standard entrophy change; and K_SV_: Stern–Volmer quenching constant.

To confirm the type of binding affinity of the complexes to CT-DNA, temperature-dependent binding constants and thermodynamic parameters were also calculated upon varying the temperature between 310 and 340 K and using spectroscopic findings (Table 2). Accordingly, the negative value for the standard Gibbs free energy change represents the spontaneous interaction of the complexes with the double helix of DNA. Furthermore, the negative ΔH^o^ and ΔS^o^ values of the Pt(tpbq)Cl_2_, Pt(ttbq)Cl_2_, and Cu(tpbq)Cl_2_ suggest their van der Waals interaction and/or hydrogen bonding, whereas the positive ΔS^o^ with the negative ΔH^o^ value of Cu(ttbq)Cl_2_ is attributed to an electrostatic interaction with CT-DNA.^47^

In the present study, several spectroscopic and viscosometric techniques were used to demonstrate the degree of DNA binding affinity of Cu(II) and Pt(II) biquinoxaline compounds. The intrinsic binding constant (K_b_) values were calculated based on the changes in the spectrum of the compounds upon the addition of CT-DNA (Fig. 3A, inset) and in accordance to Equation 2 presented in the experimental section. The Pt(II) and Cu(II) complexes with tpbq exhibited 3-fold greater affinity for CT-DNA than those containing ttbq. This might be attributed to the different donating ability of the pyridine- and thienyl-containing quinoxaline ligands to the metal ions. It was also observed that the Cu(II) tpbq and ttbq complexes maintain stronger interactions with the CT-DNA than those of Pt(II) (Table 1). These K_b_ values are even lower compared to commonly used intercalators,^43–46^ suggesting the presence of a rather weak non-covalent interaction between the compounds and CT-DNA.

The temperature-dependent binding constants were measured spectroscopically to confirm the type of binding affinity for CT-DNA in the compounds. Additionally, the thermodynamic parameters, namely the changes in the standard Gibbs free energy (ΔG^o^), standard binding enthalpy (ΔH^o^), and standard entropy (ΔS^o^) were measured according to Equations 2 and 3 with temperatures varying between 310 and 340 K, all listed in Table 1. Accordingly, the negative value for the standard Gibbs free energy change represented the spontaneous bias toward the double helix of DNA within compounds. Furthermore, the negative ΔH^o^ and ΔS^o^ values of the Pt(tpbq)Cl_2_, Pt(ttbq)Cl_2_, and Cu(tpbq)Cl_2_ suggested van der Waals and hydrogen bond interactions, whereas the positive ΔS^o^ with the negative ΔH^o^ value of Cu(ttbq)Cl_2_ was linked to electrostatic interaction with DNA.^47^

The CT-DNA was examined for viscometric behaviors in the presence of the Cu(II) and Pt(II) compounds, and the obtained values pose obvious questions concerning the nature of these processes; for instance, a rise in the DNA viscosity due to the binders can be explained by the intercalative nature of the effect as the DNA helix begins to elongate.^48^ As shown in Fig. 3B and Supplementary Figs. S20b–S22b, the viscosity and the slope of the plots related to CT-DNA slightly increase upon the action of the compounds, thus pointing to the electrostatic nature of the groove binding here. The values obtained for the relative viscosity slopes ranged from 0.0266 to 0 0.0443 but, in the case of the intercalators, this value increased up to 1.^49,50^

In the next stage, the conformational changes in the DNA helix were investigated according to the fluctuations in the electronic absorption band of the DNA at 260 nm in the presence of the compounds between 30°C and 90°C in a Tris HCl buffer. The melting temperature, *T*_m_, of DNA was measured based on the data collected during the denaturation of the double helix structure to a single-strand form by heating. The magnitude of the *T*_m_ value of DNA in the presence of any binder provides additional clues as to the nature of the binding.^51^ In light of these observations, an increase in *T*_m_ represents either an intercalative or a phosphate binding, while a decline in the temperature represents base-binding.^52^ Intercalation binding reinforces double helix formation, causing *T*_m_ to increase by 5°C to 8°C; conversely, non-intercalative binding does not cause a significant increase in *T*_m_.^53^ In the same manner, the denaturation detected on the DNA duplex triggered by the Cu(II) and Pt(II) compounds caused only minor changes in the *T*_m_ of DNA (see Fig. 3C and Supplementary Figs. S20c–S22c). The extent of change in the DNA melting temperature (Δ*T*_m_) in the presence of the complexes did not exceed 1.5°C, implying electrostatic interaction with the phosphate groups within the grooves.^48,54,55^

To shed light on the nature of the bonding in the metal-based compounds, additional fluorometric analyses were performed. To this end, we used EtBr-pretreated CT-DNA to determine the binding affinity between the compounds and DNA, later sketching a Stern–Volmer plot according to Fig. 3D and Supplementary Figs. S20d–22d. The resulting quenching constants K_SV_ for each compound ranged from 0.0082 to 0.0143 (Table 1). The K_SV_ values for CT-DNA also highlight electrostatic interaction via groove binding^56,34^ as observed in the previously obtained spectroscopic and viscometric results. Although the Cu(ttbq)Cl_2_ and Pt(ttbq)Cl_2_ complexes have a stronger binding affinity compared to their tpbq counterparts, all complexes can be said to inhibit the fluorescence of EtBr-bound CT-DNA by surface binding.^57^

### HSA-binding studies

The binding affinity of the compounds toward a plasma transport protein HSA was examined using EAS. For this purpose, the spectral changes were followed at the representative absorption band of the protein (280 nm) during UV titration, which was performed via a fixed value HSA (2.12 × 10^−5^ M) with varying concentrations of compounds (2.12 × 10^−5^ to 2.12 × 10^−4^ M).^58^

**Fig. 4:**
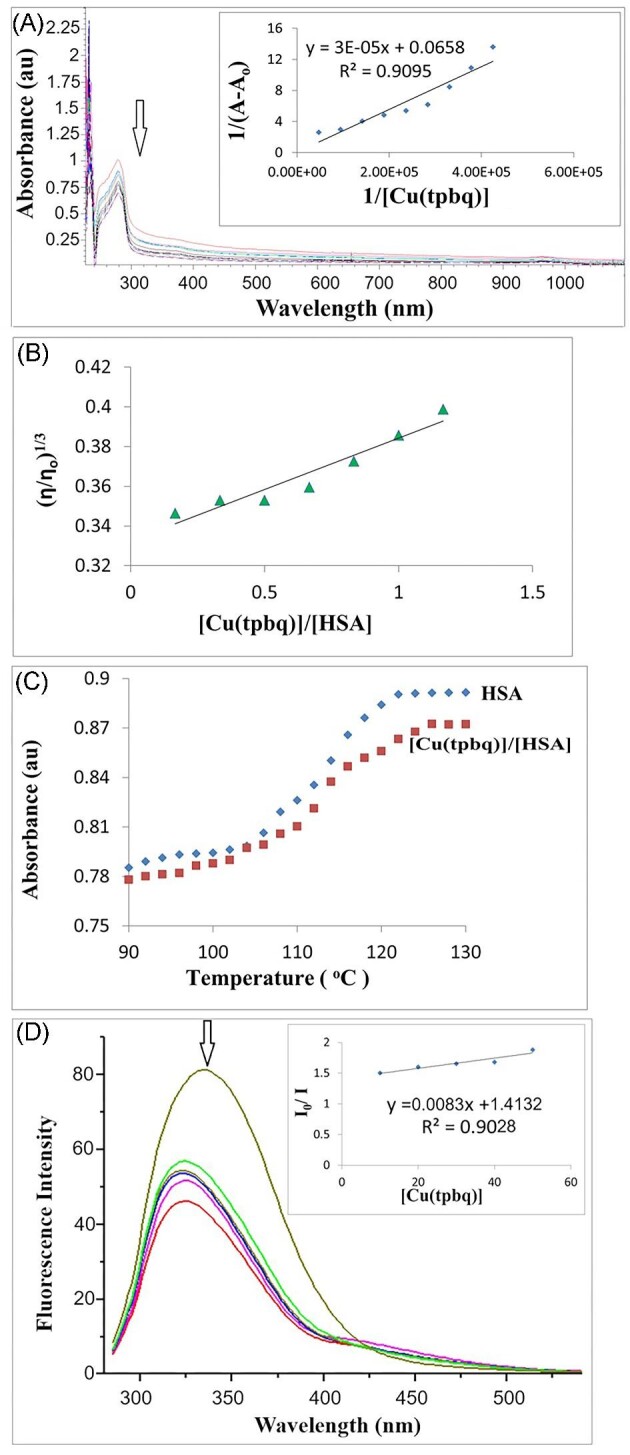
(A) Electronic absorption spectra of Cu(tpbq)Cl_2_ in the presence of HSA (2.12 × 10^5^ M) (inset: the plot used for calculating K_b_). (B) Thermal denaturation plots obtained for Cu(tpbq)Cl_2_ and HSA. (C) The changes in the relative viscosity of the HSA in the presence of Cu(tpbq)Cl_2_. (D) The change in the fluorescence spectrum of HSA in the presence of Cu(tpbq)Cl_2_ (10–50 μM) (inset: the plot used for calculating K_SV_).

The decline seen within the band intensity could be attributed to the formation of a surface adduct of compound and protein. Such formation can also imply high polarity and reduced hydrophobicity around the tryptophan residue,^59^ resulting in bonds between the compounds and the hydrophobic domains of the protein as well as a change in the HSA configuration.^60^ Conversely, an increase in absorption (hyperchromic effect) may appear if the compounds are exposed to HSA, thereby causing an adduct formation on the protein through external contact and possibly through electrostatic interactions in the protein's secondary structure. Figure 4A and Supplementary Figs. S23a–S25a demonstrate the spectral change that takes place during the titration tests. The observed hypochromic effect on the addition of compounds may be due to the hydrophobic interactions triggered by the π-π stacking relationship between the aromatic rings in the compounds and the phenyl rings at the tryptophan, tyrosine, and phenylalanine residues in the protein binding groove.^61^

The intrinsic binding constant, K_b_, is measured at about 10^4^ M^−1^ for all HSA-compound adducts (see Table 3). This result revealed the elevated affinity of all of our compounds toward HSA compared to reported complexes in other studies having binding constants between 10^5^ and 10^6^.^62^

**Table 3.** Thermodynamic data and fluorescence quenching constant values of the complexes upon binding to HSA

Compounds	K_b_(M^−1^)	ΔG^o^(J)	ΔH^o^(J)	ΔS^o^(J/K)	K_SV_
Cu(tpbq)Cl_2_	2.19 × 10^3^	−1.98 × 10^4^	12.08 × 10^4^	458.15	0.0059
Cu(ttbq)Cl_2_	4.97 × 10^4^	−2.79 × 10^4^	5.31 × 10^4^	258.51	0.0720
Pt(tpbq)Cl_2_	1.98 × 10^5^	−3.14 × 10^4^	2.29 × 10^4^	174.97	0.0261
Pt(ttbq)Cl_2_	6.67 × 10^4^	−2.86 × 10^4^	3.32 × 10^4^	198.00	0.0589

K_b_: binding constant; ΔG^o^: standard Gibbs free energy change; ΔH^o^: standard enthalpy change ΔS^o^: standard entropy change; and K_SV_: Stern–Volmer quenching constant.

Tests of UV titration at four different temperatures ranging from 310 to 340 K indicated the spontaneous affinity of the compounds toward HSA with a negative ∆G^o^ value.^63^ As the thermodynamic studies revealed in Table 2, the positive value of ΔS^o^ and ΔH^o^ imply the presence of entropy-driven electrostatic coupling interactions between compounds and HSA.^64^

Viscosity tests are another useful tool for determining the mode of action between Cu(II) and Pt(II) compounds and albumin. The electrostatic interactions between these compounds and HSA are likely to increase the relative viscosity of the mixture.^65^ Minor changes in the related figures can be observed upon the addition of compounds that form bonds in the HSA groove.^66^ Binding with HSA can be attributed to hydrophobic processes if there is an insignificant increase in the relative specific viscosity with increasing compound concentrations.^67^ Figure 4B and Supplementary Figs. S23b–S25b show changes in the relative viscosity of HSA in the presence of compounds. A small increase (0.0495–0.0657) in the slope of the graph for relative viscosity is indicative of interactions in the hydrophobic domain.^68^

Thermal denaturation of proteins is a major challenge in their separation/repositioning, biotransformation, and biosensing as well as drug production and food production.^69^ Generally, when a compound binds to a protein in its native state, this causes the temperature to remain constant and the corresponding index improves. However, the thermal denaturation analyses conducted here indicate that the melting point of HSA declined from 114°C to 110°C, 111°C, 109°C, and 105°C upon the addition of Cu(tpbq)Cl_2_, Cu(ttbq)Cl_2_, Pt(tpbq)Cl_2_, and Pt(ttbq)Cl_2_, respectively (Fig. 4C, Supplementary Figs. S23c–S25c). Consequently, it can be stated that once albumin binds with our complexes, it loses its stability.

Fluorescent quenching assessments for albumin are crucial as they can reveal the interactions that HSA can have with any drug.^70^ Quite commonly, fluorescence emerges thanks to three inherent properties: tryptophan, tyrosine, and phenylalanine residues of HSA. Under real conditions, pure fluorescence in most proteins is mainly mediated by tryptophan alone Nonetheless, given the very low quantum yields of phenylalanine, the fluorescence of a tyrosine can be completely eliminated by ionization.^71^ The changes in the emission band of HSA at 346 nm were also monitored thoroughly after the compounds were administered to obtain information about how the compounds affect the structural conformation of serum albumin. On this basis, it became evident that our complexes reduced the emission intensity without any significant shifts in the peak position; thereby, suggesting that they interacted with HSA by producing non-fluorescent adducts.^72^ In other words, the Cu(II) and Pt(II) complexes interacted with serum albumin via the hydrophobic region located inside the protein (Fig. 4D, Supplementary Figs. S23d–S25d).^73^

### Cytotoxicity of Cu(II) and Pt(II) complexes

**Fig. 5:**
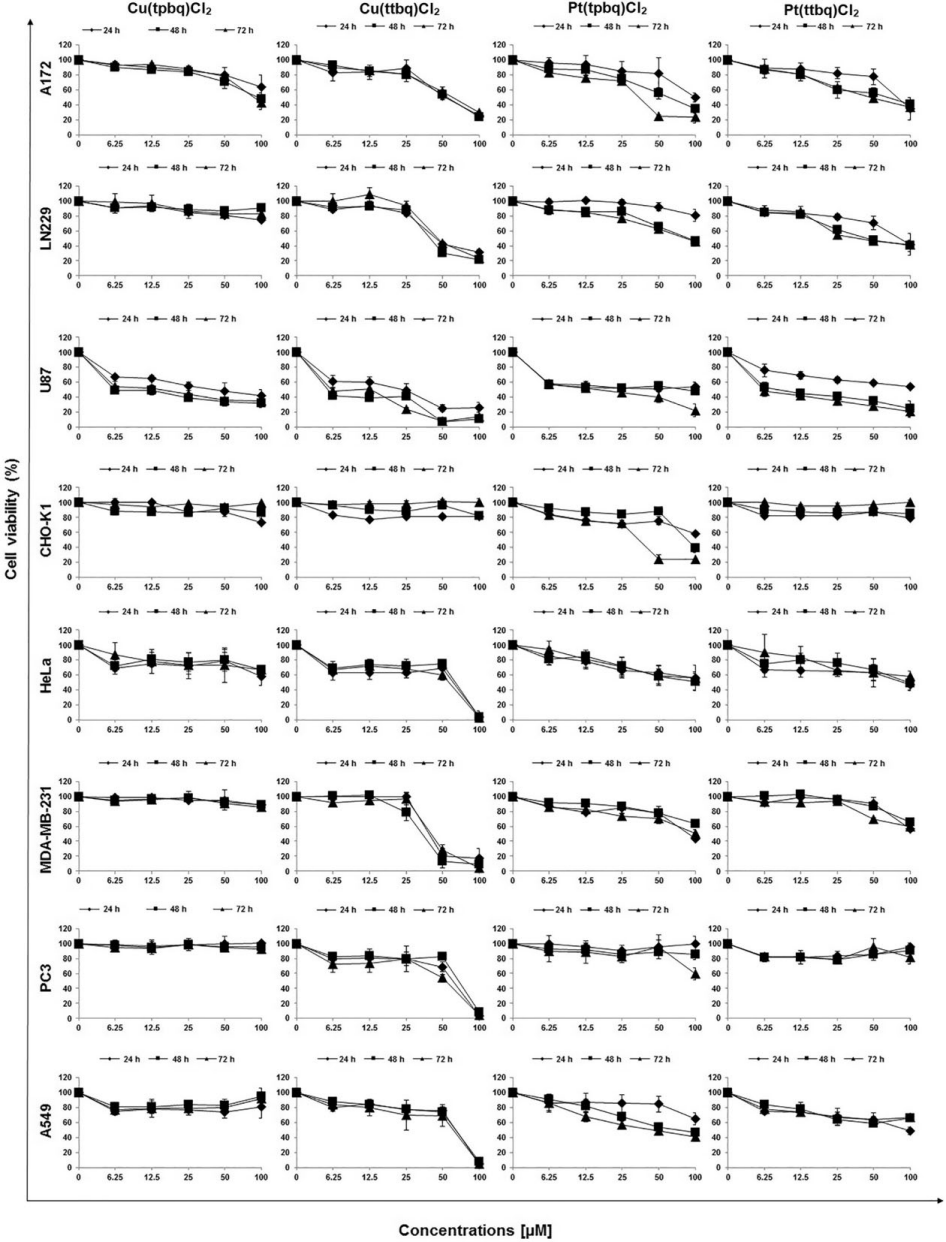
The dose–response curves for the Cu(II) and Pt(II) complexes using cell lines from different origins (A172, LN229, U87, A549, HeLa, MDA-MB-231, PC3, CHO-K1) obtained upon exposure for 24, 48, and 72 h using MTT cell viability assay.

The human cancer cell lines, glioblastoma (A172, LN229, U87), cervix (HeLa), breast (MDA- 231), lung (A-549), prostate (PC-3) and a non-cancer Chinese hamster ovary CHO-K1 cell line as a control were used to determine the cytotoxicity of the Cu(tpbq)Cl_2_, Cu(ttbq)Cl_2_, Pt(tpbq)Cl_2_ and Pt(ttbq)Cl_2_ complexes by using an MTT assay. The dose–response relationship between the examined Cu(II) and Pt(II) complexes and the used cell lines were obtained from the percentage cell viability vs. exposed concentrations (Fig. 5). The 50% inhibition concentration (IC_50_) for each compound for 24, 48, and 72 h exposure period was obtained from the dose–response curve as presented in Table 4. Overall, Cu(ttbq)Cl_2_ was found to have the most anticancer potential among the examined complexes across the tested cell lines, followed by Pt(tpbq)Cl_2_ > Pt(ttbq)Cl_2_ > Cu(tpbq)Cl_2_ in a descending order. The cytotoxic effects of the Cu(II) and Pt(II) complexes did not increase parallel to their derivatives; in another words, the ttbq derivative of Cu(II) was more cytotoxic than the tpbq derivatives. Conversely, the tpbq derivative of Pt(II) was more cytotoxic than the ttbq derivatives. The most sensitive cancer cells upon exposure to the complexes were found to be U87 and HeLa cell lines, which were set aside for use in other assays. The MDA231 cell line was also seen to be sensitive, but only against Cu(ttbq)Cl_2_ and not any other complex. As expected, the non-cancer control, CHO-K1, cell line was less responsive than the others.

**Table 4.** TheIC_50_ values of the synthesized Cu(II) and Pt(II) complexes calculated from the dose–response curves obtained using MTT cell viability assay and cell lines from a variety of origins (A172, LN229, U87 A549, Hela, MDA231, PC3 and CHO-K1) upon exposure for 24, 48, and 72 h

		IC_50_ values, μM			
Cell lines	Exposure (h)	Cu(tpbq)Cl_2_	Cu(ttbq)Cl_2_	Pt(tpbq)Cl_2_	Pt(ttbq)Cl_2_
A172	24	>100	69.44 ± 0.19	94.29 ± 12.27	93.50 ± 18.37
	48	>100	63.88 ± 3.56	39.38 ± 15.82	65.52 ± 17.73
	72	92.04 ± 7.46	57.41 ± 13.68	73.67 ± 0.28	49.47 ± 5.73
LN229	24	>100	42.04 ± 13.99	>100	67.86 ± 16.32
	48	>100	36.00 ± 6.38	91.89 ± 049	44.78 ± 11.61
	72	>100	54.01 ± 3.1	83.85 ± 3.16	38.76 ± 1.46
U87	24	79.46 ± 26.38	31.18 ± 0.43	58.35 ± 11.81	>100
	48	5.49 ± 0.01	2.95 ± 0.97	34.68 ± 2.16	9.24 ± 4.16
	72	13.53 ± 6.06	5.75 ± 0.03	22.09 > 10016.24	5.95 ± 1.68
A549	24	>100	73.55 ± 1.35	>100	95.02 ± 2.93
	48	>100	70.68 ± 4.09	58.44 ± 5.35	>100
	72	>100	66.90 ± 8.70	40.26 ± 3.42	>100
HeLa	24	>100	67.52 ± 2.78	>100	88.85 ± 8.31
	48	>100	74.22 ± 0.45	57.97 ± 26.86	79.69 ± 24.21
	72	>100	64.99 ± 2.75	42.86 ± 1.69	>100
MDA231	24	72.14 ± 26.04	20.59 ± 2.39	94.81 ± 2.57	85.00 ± 10.81
	48	>100	32.11 ± 1.05	>100	>100
	72	>100	40.95 ± 0.60	99.37 ± 5.71	>100
PC3	24	>100	65.75 ± 5.35	>100	>100
	48	>100	75.50 ± 3.16	>100	>100
	72	>100	62.31 ± 4.02	>100	>100
CHO-K1	24	>100	>100	>100	>100
	48	>100	>100	86.63 ± 1.98	>100
	72	>100	>100	77.52 ± 0.87	>100

In another study, novel Cu(II)-2-(2′-pyridyl) quinoxaline complexes were found more cytotoxic on human breast (MCF-7) and human embryonic kidney (HEK-293) cell lines compared to the cisplatin used as a positive control.^74^ The Cu-complex was also evaluated by another study group against six different cancer cell lines (HL-60 cells, PC-3M-1E8 human prostate tumor cells, BGC-832 cells, MDA cells, Bel-7402 human hepatoma cells, and Hela human cervix cancer cells), showing a considerable inhibitory rate and cytotoxic specificity.^75^ Another study investigated Pt(II)-terpyridine complexes, which were found to be strongly cytotoxic to human cancer cell lines HCT116 (colorectal), SW480 (colon), NCI-H460 (non-small cell lung), and SiHa (cervix). Their IC_50_ values ranged from 0.05 to 4.4 μM.^76^ In a different research, several platinum-based complexes, owning various types of ligands and revealing excellent anticancer activity, showed higher cytotoxic effects against MCF-7, A549, and HCT-116 cell lines in comparison with clinically used cisplatin and oxaliplatin.^77^ Overall, these previous studies and our study on Cu(II) and Pt(II) complexes demonstrate high anticancer potential against cancer cell lines from a variety of origins.

### ROS production—DCFDA assay

**Fig. 6:**
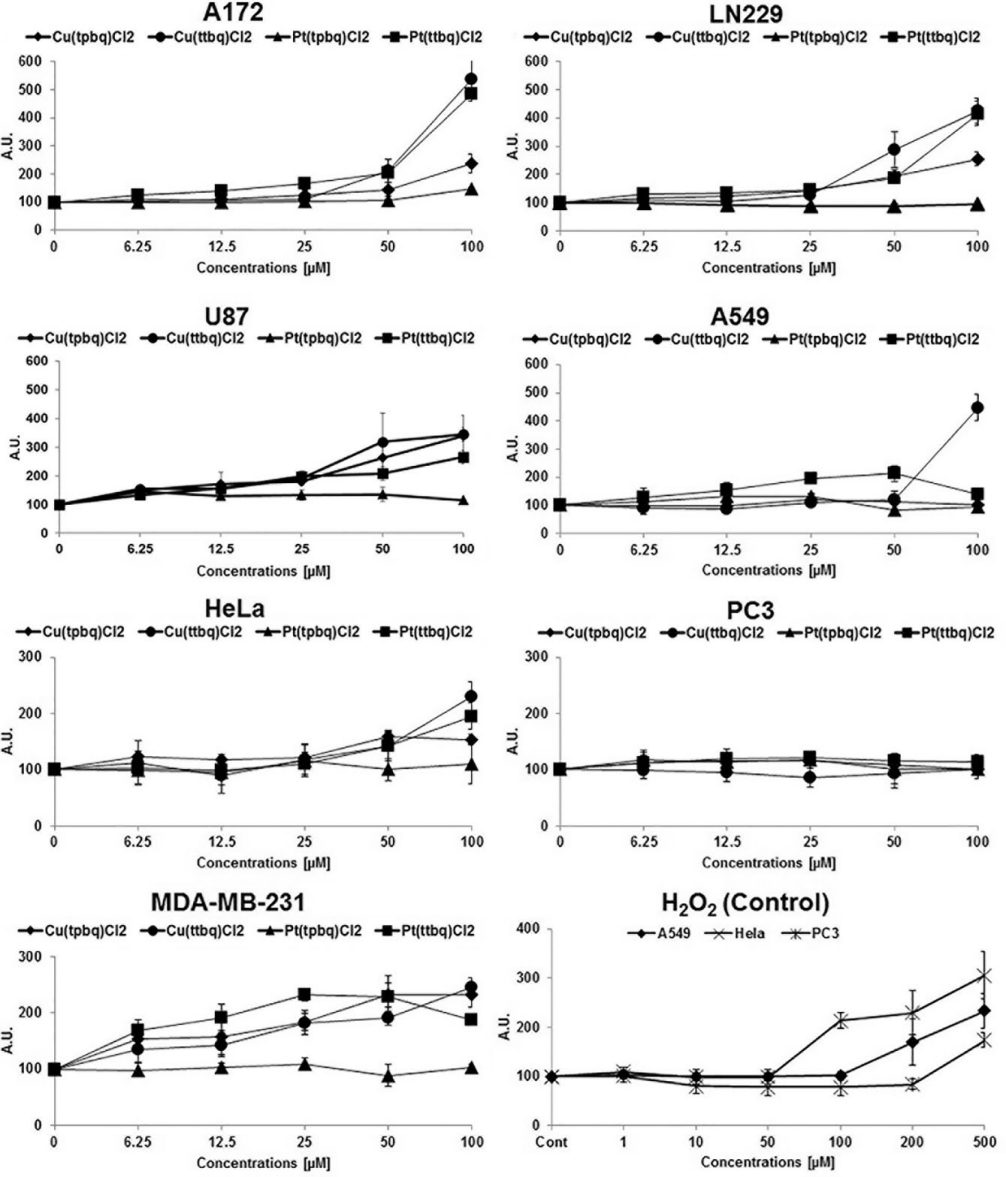
The ROS generation of the used cell lines from different origins (A172, LN229, U87 A549, Hela, PC3, and MDA231) upon exposure to the Cu(II) and Pt(II) complexes for 24 h in the DCFDA assay. H_2_O_2_ was used as a positive control at the indicated doses.

Oxidative stress in an alive system is generally measured by using the fluorescent marker dihydrodichlorofluorescein diacetate (H(2)DCF-DA), which is de-esterified and oxidized to fluorescent DCF (2ʹ,7ʹ-dichlorofluorescein).^78,79^ In order to determine DCFDA activity that increases with increased ROS generation in response to oxidative stress, ROS generation is determined as a DCFDA signal for a particular condition referring to the corresponding proliferation counts. In this study, the Cu(II) and Pt(II) complexes at concentrations from 6.25 to 100 μM were administered to the used A172, LN229, U87, A549, Hela, PC-3, and MDA-231 cell monolayers for 24 h and, then, the ROS formation was measured using a DCFDA fluorescence agent with a fluorescence spectrophotometer. Figure 6 shows a comparison of the level of ROS generation in the cells against the negative control including growth medium in response to the synthesized complexes. As a result of this assay, the dithenyl derivatives of Cu(II) and Pt(II) complexes generated more ROS than the dipyridyl derivatives on the used cell lines. Overall, Cu(ttbq)Cl_2_ caused the highest increase in the level of ROS formation, followed by Pt(ttbq)Cl_2_ and Cu(tpbq)Cl_2_, in the used cell lines. To elaborate, Cu(ttbq)Cl_2_ and Pt(ttbq)Cl_2_ increased ROS production 3- to 5-fold in human glioblastoma (A172, LN229, U87) and almost 2-fold in the lung A549, cervix HeLa, and breast MD-A231 cells, while no formation in prostate PC3 cells. Furthermore, Pt(tpbq)Cl_2_ did not induce any ROS formation on any of the used cell lines. Human glioblastoma cell lines produced more ROS than the other used cell lines. H_2_O_2_ was tested as a positive control and caused 3-fold ROS generation at 500 μM concentration. It was reported earlier in another study that the five binuclear Pt(II) complexes enhanced oxidative stress formation in fibroblasts by means of increased ROS generation and decreased antioxidant property.^80^

### Apoptosis—TUNEL assay

**Fig. 7:**
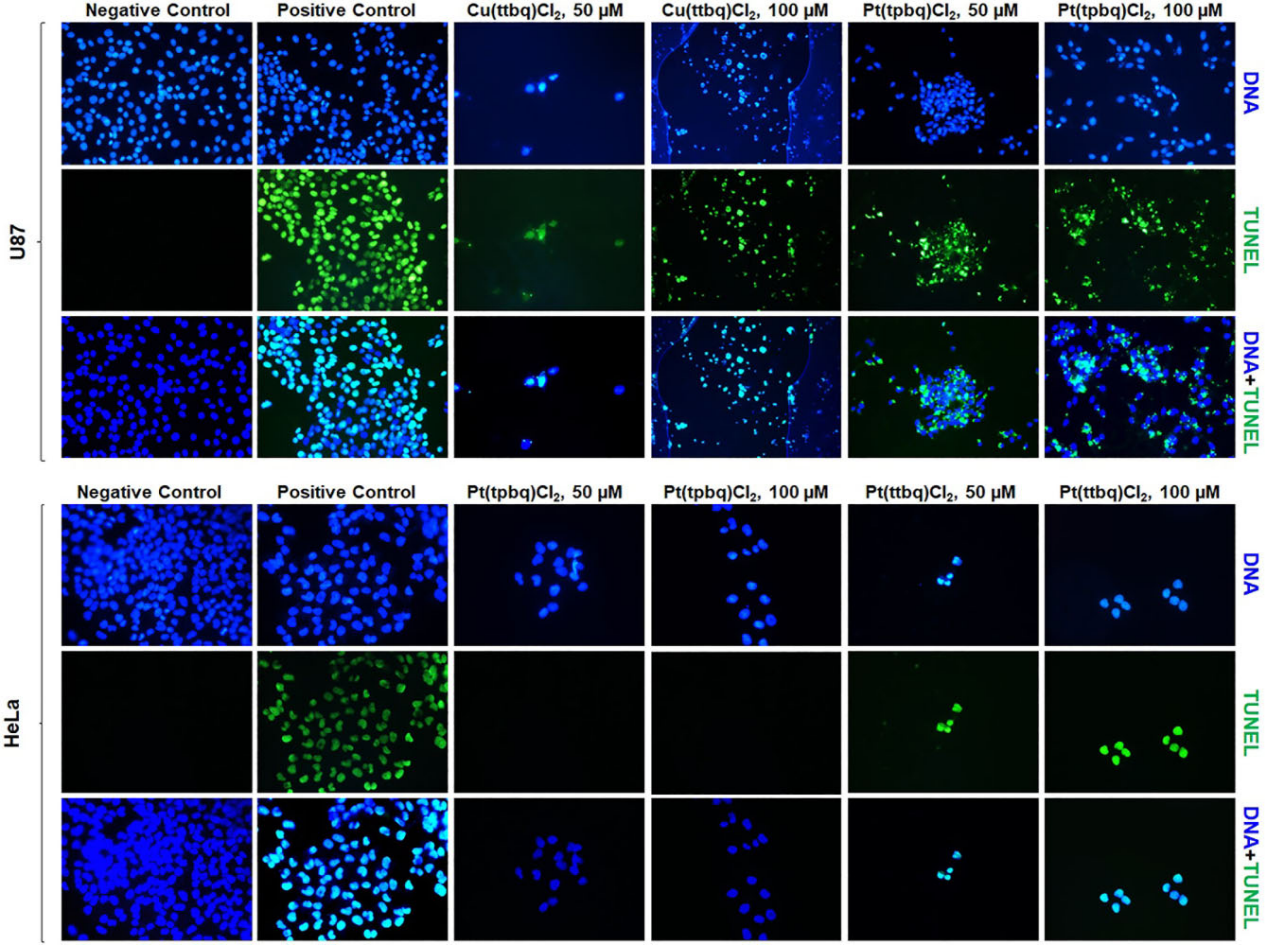
The apoptotic potency of the U87 and HeLa cells treated with the Cu(II) and Pt(II) complexes at concentrations of 50 and 100 μM for 24 h evaluated by applying TUNEL assay. The Negative control was treated with only a growth medium, and the positive control was treated with Dnase. The images were taken with 40× magnification using a fluorescence microscope.

Apoptosis is one of the well-known main cell death patterns which is a cellular suicide process triggered by specific proteins.^81^ The effects of the synthesized Cu(II) and Pt(II) complexes on the apoptotic potential of the U87 and HeLa cell lines were investigated by performing the TUNEL assay. It was designed to detect apoptotic cells that undergo extensive DNA fragmentation during the late stages of apoptosis resulted from the activation of endonucleases. The DNA fragmentation patterns of the sensitive U87 and HeLa cell lines were analysed upon exposure to Cu(II) and Pt(II) complexes at concentrations of 50 and 100 μM for 24 h (Fig. 7). The apoptotic potential of the U87 cells was observed after Cu(ttbq)Cl_2_ and Pt(tpbq)Cl_2_ treatments at both concentrations. On the other hand, apoptosis on HeLa cells was only observed upon exposure to the Pt(ttbq)Cl_2_. Cu(tpbq)Cl_2_, and Pt(ttbq)Cl_2_ on the U87 cells, and Cu(ttbq)Cl_2_, Cu(tpbq)Cl_2_, and Pt(tpbq)Cl_2_ on the HeLa cells, none of which induced any apoptosis upon 24 h treatment (data not presented here). Indeed, DNA fragmentation at a higher concentration (100 μM) compared to one obtained at a low concentration (50 μM) was higher in both cell lines (U87 and HeLa). A similar study has shown that a novel series of pyrazole-platinum(II) complexes as potential anticancer agents induced cell cycle arrest and apoptosis in breast cancer MCF-7 and MDA-MB-231 cells.^82^

### *In vitro* cell invasion and migration assays

Cancer cells must migrate and invade through the extracellular matrix to enter the bloodstream, attach to a distant site, and spread out of a vessel. Cell migration studies related to metastatic progression have gained importance in cancer research since this progression is considered as the main cause of death in patients. Tumor metastasis, the migration and invasion of tumor cells, is one of the biggest obstacles in anticancer treatment.^83,84^  *In vitro* cell invasion assay is a widely used method to provide an assessment of cell invasive capacity. In the assay used for this study, the Corning Matrigel matrix acts as an *in vitro* membrane that prevents noninvasive cells from migrating, but invading cells (malignant and nonmalignant) enzymatically degrade the matrix and spread through the membrane pores.^85^

**Fig. 8:**
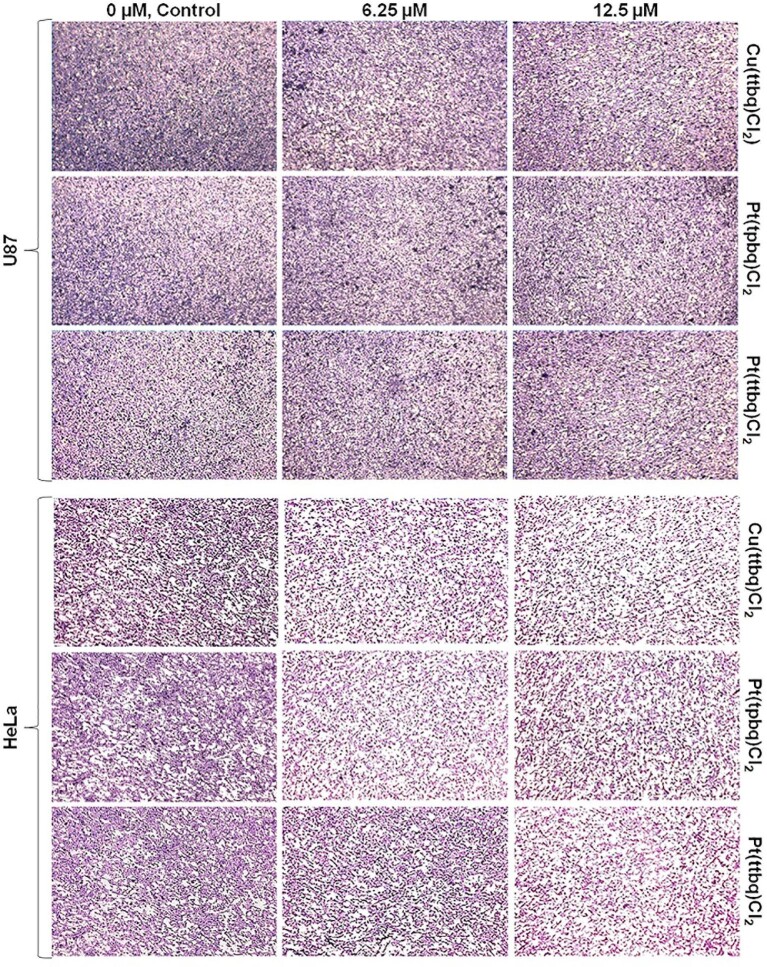
The anti-invasive effects of Cu(ttbq)Cl_2_, Pt(tpbq)Cl_2_, and Pt(ttbq)Cl_2_ at concentrations of 6.25 and 12.5 μM on U87 and HeLa cells across the Matrigel during 24 h treatment by Matrigel invasion assay as compared with the negative control. The images were taken with 40× magnification.

The anti-invasive capacity of the synthesized Cu(II) and Pt(II) complexes on the U87 and HeLa cells across the Matrigel matrix was observed under microscope (Fig. 8).

The Cu(ttbq)Cl_2_ and Pt(tpbq)Cl_2_ complexes at concentrations of 6.25 and 12.5 μM showed high anti-invasive effects on the HeLa cell monolayer as compared with the negative control, but not on the U87 cell monolayer. As seen in Fig. 8, there were no differences between the anti-invasive capacity of either concentrations of the Cu(ttbq)Cl_2_ and Pt(tpbq)Cl_2_ complexes on the HeLa cells, except Pt(tpbq)Cl_2_ which was only effective at the high concentration (12.5 μM). The Cu(tpbq)Cl_2_ complex did not have any anti-invasive effect on either of the cell lines (data not presented here). Similarly, Gu *et al.* reported that copper compounds inhibited the invasion abilities of HeLa cells, whereas, there was no significant difference found in the U87 cell line.^86^

**Fig. 9:**
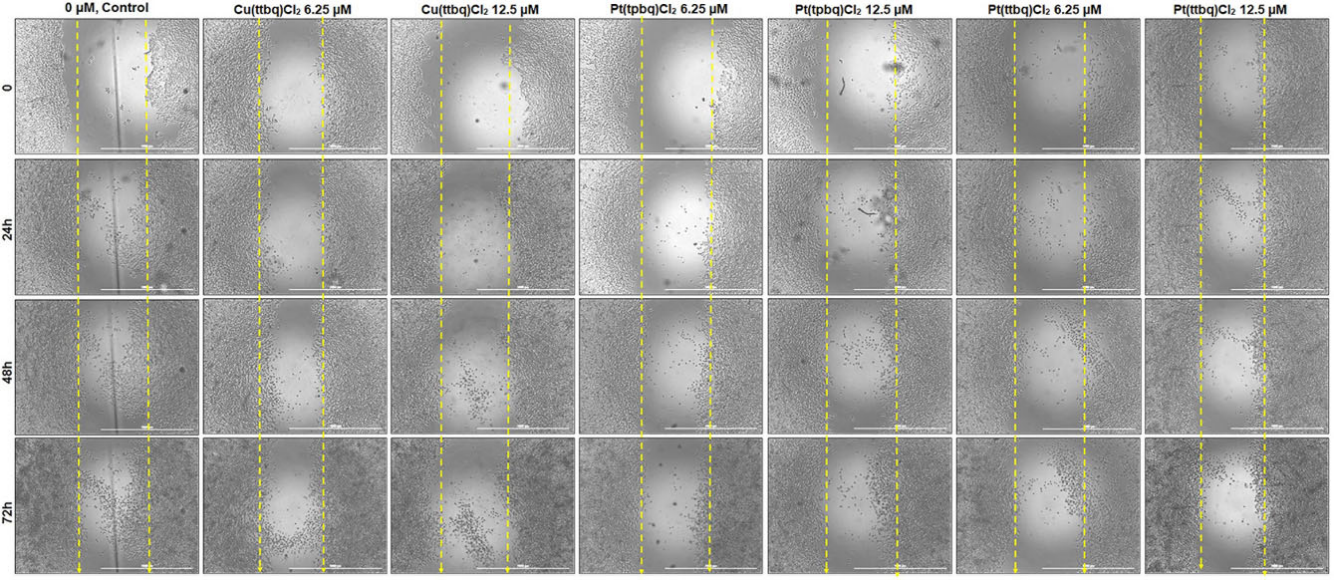
An *in vitro* cell migration assay using a human cervix HeLa cell line after 24 h treatment with Cu(ttbq)Cl_2_, Pt(tpbq)Cl_2_, and Pt(ttbq)Cl_2_ at concentrations of 6.25 and 12.5 μM. The images were acquired at 0, 24, 48, and 72 h with an inverted microscope.

The scratch wound healing assay is widely used for screening novel anticancer candidate drugs. It may provide useful information to understand how well a particular cell type can spontaneously migrate or respond to a chemo-attractant and directionally migrate toward it. As a result of the cell invasion assay, Hela was found the most sensitive cell line against the tested compounds; hence, its selection to use for the cell migration assay. The potential inhibitory effects of Cu(II) and Pt(II) complexes on the migration of HeLa are presented in Fig. 9.

The number of migrating HeLa cells toward the scratch zone upon treatment by Cu(ttbq)Cl_2_, Pt(ttbq)Cl_2_, and Pt(tpbq)Cl_2_ at concentrations of 6.25 and 12.5 μM declined during 24, 48, and 72 h treatments. However, the inhibitory effects of the tested complexes did not improve with increasing concentrations. As a result of the cell migration assay, the examined Cu(II) and Pt(II) complexes were shown to have an anti-migration potential on the HeLa cell line, and they could serve as anti-cancer drug candidates for further investigation.

### Genotoxicity

#### *In vitro* comet assay

**Fig. 10:**
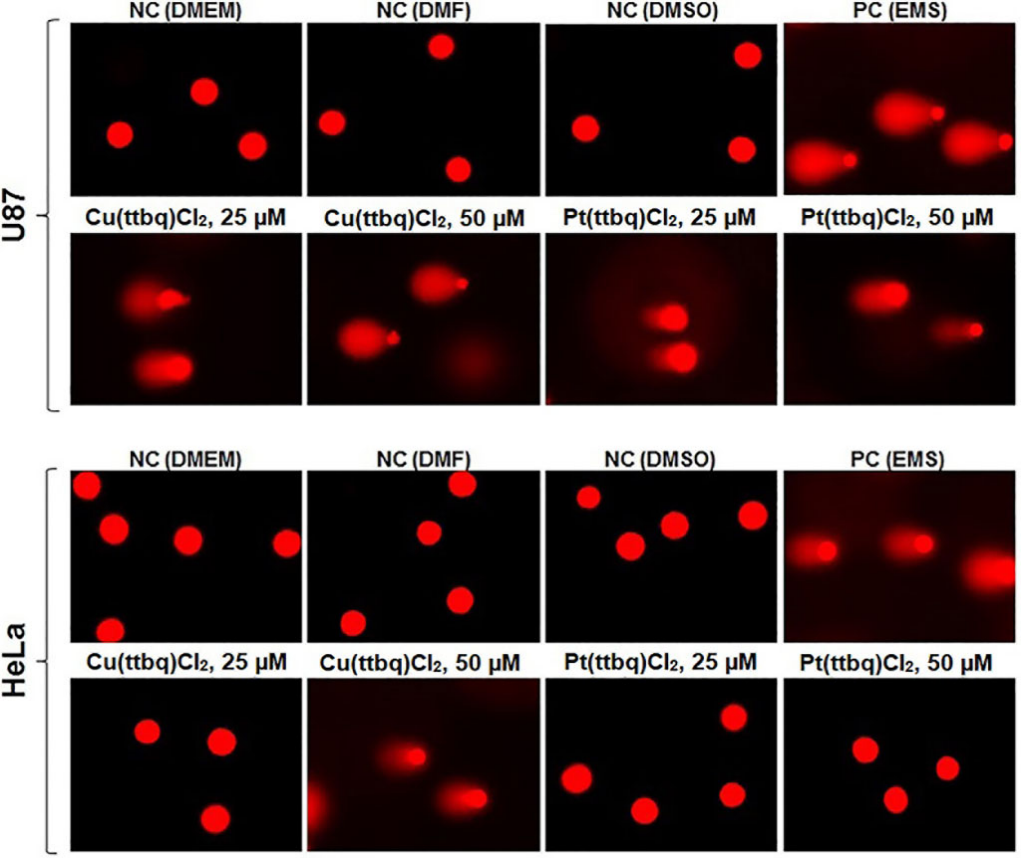
Single-cell gel electrophoresis of U87 and HeLa cell lines following treatment with the Cu(II) and Pt(II) complexes for 24 h. Negative control: growth medium; Positive control: ethyl methane sulfonate (EMS)-treated cells. Representative microscopic images of cells were taken at 40× magnification with fluorescence microscope.

To assess DNA damage, a crucial confounding factor in defining the genotoxic potential of the synthesized complexes was investigated by means of a comet assay, which precisely detected the DNA damage and revealed the genotoxic potential.^87^ The effective anticancer potential of the Cu(ttbq)Cl_2_ and Pt(ttbq)Cl_2_ complexes was determined from previous assays. Therefore, their genotoxicity at concentrations of 25 and 50 μM was examined on the HeLa and U87 cell lines using an *in vitro* comet assay. The results demonstrated that Cu(ttbq)Cl_2_ and Pt(ttbq)Cl_2_ caused DNA damage on the U87 cells at both concentrations; whereas, Cu(ttbq)Cl_2_ was only genotoxic on the Hela cells at a high concentration (50 μM), while Pt(ttbq)Cl_2_ was not genotoxic on either of the used cell lines. The DNA fragmentation on the U87 cell line upon Cu(ttbq)Cl_2_ and Pt(ttbq)Cl_2_ treatments for 24 h increased with elevated concentrations. Briefly, Cu(ttbq)Cl_2_ showed more genotoxic potential than the other synthesized complexes—a finding that is supported by the results obtained for other assays in this study (Fig. 10).

#### Plasmid DNA interaction assay

**Fig. 11:**
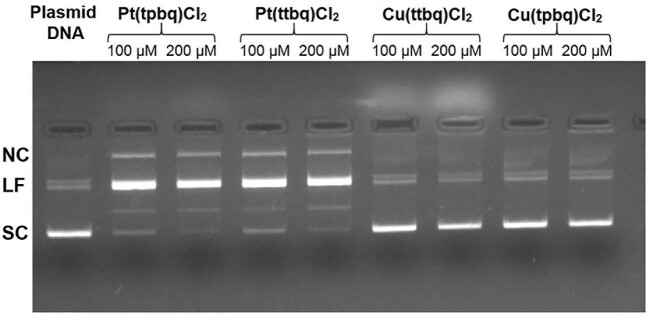
Plasmid DNA interaction assay with pBI-CMV1 plasmid (3.1 kb) grown in *Escherichia coli* and, then, purified using a Machery Nagel DNA isolation kit. The plasmid DNA incorporation after treatments with 100 and 200 μM concentrations of the Cu(II) and Pt(II) compounds were analysed, and the images of the bands were captured using a ChemiDoc imaging system (BioRad). The testing was done in two repeats. SC: Supercoiled; NC: Nicked circular; LF: Linear form

The genotoxic effects of the synthesized Cu(II) and Pt(II) complexes at concentrations of 100 and 200 μM were analysed with plasmid DNA interaction assay. The migration pattern of the treated plasmid DNA with the Cu(II) and Pt(II) complexes was evaluated by agarose gel electrophoresis. The pure plasmid DNA as a negative control in the absence of any external agents was examined and the change in migration pattern was evaluated in the gel (Fig. 11). The DNA cleavage activities of the complexes were evaluated by determining their ability to convert the SC plasmid DNA to open circular form and linear form. The pure plasmid DNA is mainly composed of SC and NC forms. The conversion process mainly led to a decline in the intensity of the NC bands, possibly due to the accumulation of too many nicks leading to the degradation of SC-DNA. Pt(II) treatment led to plasmid DNA degradation, which was observed in the increased intensity of NC bands and linear form. On the other hand, the Cu(II) complexes did not cause any plasmid DNA degradation in the agarose gel.

Although the synthesized Cu(II) complexes did not interact with plasmid DNA in this study, previous studies have shown that other copper complexes may act by different mechanisms, such as ROS generation, which bring about oxidative cell damage and consequently trigger cancer cell death through an apoptotic mechanism.^88^ Indeed, in our study, synthesized Cu(II) complexes caused an increase in the level of ROS generation.

Similar results have been previously reported. For instance, novel Cu(II)-2-(2ʹ-pyridyl) quinoxaline complexes to cause DNA cleavage and become more cytotoxic on human breast MCF-7 and human embryonic kidney HEK-293 cell lines compared to cisplatin used as a positive control.^74^ Likewise, another study on platinum(II)-quinoxaline compounds revealed that they interacted with calf-thymus DNA and demonstrated high levels of cytotoxic effect on L1210 murine leukemia cell lines.^89^

## Conclusion

In the present study, Cu(II) and Pt(II) complexes of tpbq and ttbq were synthesized and characterized. The molecular structure of the complexes was determined theoretically. Calculations at the B3LYP/LANL2DZ level of theory showed that Cu(tpbq)Cl_2_ and Pt(tpbq)Cl_2_ display a deviation from the square planar structure around the metal center. This deviation was significant in Cu(tpbq)Cl_2_, while slight in Pt(tpbq)Cl_2_. The optimized geometry calculations for Cu(ttbq)Cl_2_ and Pt(ttbq)Cl_2_, on the other hand, exhibited a coordination between the metal center and an H-atom on the quinoxaline, as well as a nitrogen atom coordination of the same unit. The interaction of the metal ions with the H-atom led to a deviation from planarity.

The nature of the binding of the complexes on DNA and HSA were determined as electrostatic and hydrophobic interactions, respectively.

The cytotoxicity of the complexes indicated that Cu(ttbq)Cl_2_ and Pt(tpbq)Cl_2_ were the most effective complexes, and the U87 and HeLa cell lines were determined to be the most effected cell lines upon exposure to the four compounds. DNA damage was generated by compounds dependent on ROS formation, and the cell death pattern was identified as apoptosis for the U87 and HeLa cell lines upon the addition of Cu(ttbq)Cl_2_, Pt(tpbq)Cl_2_, and Pt(ttbq)Cl_2_.

Lastly, the Cu(ttbq)Cl_2_, Pt(tpbq)Cl_2_, and Pt(ttbq)Cl_2_ complexes demonstrated higher potency to prevent the invasion and migration of HeLa cells. Thus, in accordance with our findings, Cu(ttbq)Cl_2_, Pt(tpbq)Cl_2_, and Pt(ttbq)Cl_2_ have a higher potential to serve as anticancer drug candidates and deserve further and more comprehensive studies.

## Supplementary Material

mfae001_Supplemental_File

## Data Availability

The data underlying this article will be shared on reasonable request to the corresponding author.
