# Supplementary material for: Antiproliferative activity of platinum(II) and copper(II) complexes containing novel biquinoxaline ligands
Source: Metallomics. 2024 Jan 5;16(2):mfae001. doi: 10.1093/mtomcs/mfae001 (PMC10849753; doi:10.1093/mtomcs/mfae001)
Supplement: mfae001_Supplemental_File [file mfae001_supplemental_file.docx]

***SUBLEMENTARY INFORMATION***

**Antiproliferative Activity of Platinum(II) and Copper(II) Complexes Containing Novel Biquinoxaline Ligands**

**Hager Sadek El –Beshti^a^, Zuhal Gercek^b^, Hakan Kayi^c^, Yasemin Yildizhan^d^, Yuksel Cetin^d^, Zelal Adigüzel^e^, Gamze Güngör^d^, Şeniz Özalp-Yaman^a,*^**

**Figure S1.** Mass spectrum of tpbq_._

**Figure S2.** Mass spectrum of ttbq_._

**Figure S3**. Mass spectrum of [Cu(tpbq)Cl_2_]

**Figure S4.** Mass spectrum of [Cu(ttbq)Cl_2_]

**Figure S5.** Mass spectrum of [Pt(tpbq)Cl_2_]

**Figure S6.** Mass spectrum of [Pt(ttbq)Cl_2_].

**Figure S7.** ^1^H-NMR spectrum of tpbq in CDCl_3_

**Figure S8.** ^1^H-NMR spectrum of ttbq in CDCl_3_

**Figure S9.** ^13^C -NMR spectrum of tpbq in d_6_-DMSO

**Figure S10**. ^13^C -NMR spectrum of ttbq in d_6_-DMSO

**Figure S11.** ^1^H-NMR spectrum of [Cu(tpbq)Cl_2_] complex in d_6_-DMSO.

**Figure S12.** ^1^H-NMR spectrum of [Cu(ttbq)Cl_2_] complex in d_6_-DMSO.

**Figure S13.** ^1^H-NMR spectrum of [Pt(tpbq)Cl_2_] complex in d_6_-DMSO.

**Figure S14.**  ^1^H-NMR spectrum of [Pt(ttbq)Cl_2_] complex in d_6_-DMSO.

**Figure S15.** Raman spectrum of [Cu(tpbq)Cl_2_].

**Figure S16.** Raman spectrum of [Cu(ttbq)Cl_2_].

**Figure S17.** Raman spectrum of [Pt(tpbq)Cl_2_].

**Figure S18**. Raman spectrum of [Pt(ttbq)Cl_2_].

**Figure S19:** Electronic absorption spectrum of (a) Cu(tpbq)Cl_2_, (b) Cu(ttbq)Cl_2_, (c) Pt(tpbq)Cl_2_ and (d) Pt(ttbq)Cl_2_.

**Figure S20.** (a) Electronic absorption spectra of Cu(ttbq)Cl_2_ in the absence and in the presence of increasing amount of CT-DNA (0-7.80x 10^-4^) (inset: the plot used for calculating K_b_). (b) Thermal denaturation plots obtained for Cu(ttbq)Cl_2_ and CT-DNA. (c) The changes in the relative viscosity of the CT-DNA in the presence of Cu(ttbq)Cl_2_. (d) The change in the Fluorescence spectrum of EtBr-bound CT-DNA in the presence of Cu(ttbq)Cl_2_ (10-160 μM) (inset: the plot used for calculating K_SV_).

**Figure S21.** (a) Electronic absorption spectra of Pt(tpbq)Cl_2_ in the absence and in the presence of increasing amount of CT-DNA (0 -5.02x 10^-4^) (inset: the plot used for calculating K_b_). (b) Thermal denaturation plots obtained for Pt(tpbq)Cl_2_ and CT-DNA. (c) The changes in the relative viscosity of the CT-DNA in the presence of Pt(tpbq)Cl_2_. (d) The change in the Fluorescence spectrum of EtBr-bound CT-DNA in the presence of Pt(tpbq)Cl_2_ (10-160 μM) (inset: the plot used for calculating K_SV_).

**Figure S22.** (a) Electronic absorption spectra of Pt(ttbq)Cl_2_ in the absence and in the presence of increasing amount of CT-DNA (0- 5.86x 10^-3^) (inset: the plot used for calculating K_b_). (b) Thermal denaturation plots obtained for Pt(ttbq)Cl_2_ and CT-DNA. (c) The changes in the relative viscosity of the CT-DNA in the presence of Pt(ttbq)Cl_2_. (d) The change in the Fluorescence spectrum of EtBr-bound CT-DNA in the presence of Pt(ttbq)Cl_2_ (10-160 μM) (inset: the plot used for calculating K_SV_).

**Figure S23.** (a) Electronic absorption spectra of Cu(ttbq)Cl_2_ in the presence amount of HSA (2.12x10^5^ M) (inset: the plot used for calculating K_b_). (b) Thermal denaturation plots obtained for Cu(ttbq)Cl_2_ and HSA. (c) The changes in the relative viscosity of the HSA in the presence of Cu(ttbq)Cl_2_. (d) The change in the fluorescence spectrum of HSA in the presence of Cu(ttbq)Cl_2_ (10-50 μM) (inset: the plot used for calculating K_SV_).

**Figure S24**. (a) Electronic absorption spectra of Pt(tpbq)Cl_2_ in the presence amount of HSA (2.12x10^5^ M) (inset: the plot used for calculating K_b_). (b) Thermal denaturation plots obtained for Pt(tpbq)Cl_2_ and HSA. (c) The changes in the relative viscosity of the HSA in the presence of Pt(tpbq)Cl_2_. (d) The change in the fluorescence spectrum of HSA in the presence of Pt(tpbq)Cl_2_ (10-50 μM) (inset: the plot used for calculating KSV).

**Figure S25**. (a) Electronic absorption spectra of Pt(ttbq)Cl_2_ in the presence amount of HSA (2.12x10^5^ M) (inset: the plot used for calculating K_b_). (b) Thermal denaturation plots obtained for Pt(ttbq)Cl_2_ and HSA. (c) The changes in the relative viscosity of the HSA in the presence of Pt(ttbq)Cl_2_. (d) The change in the fluorescence spectrum of HSA in the presence of Pt(ttbq)Cl_2_ (10-50 μM) (inset: the plot used for calculating K_SV_).

**
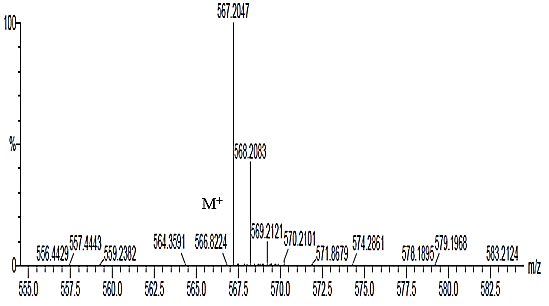
**

**Figure S1**

**
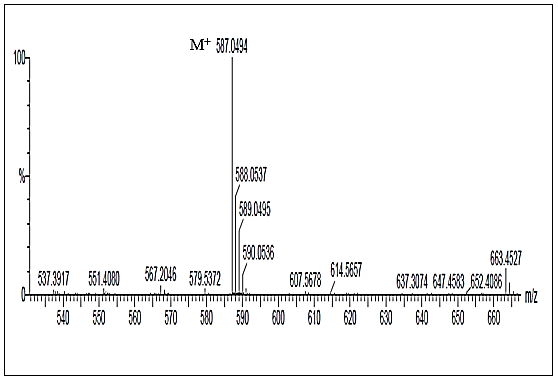
**

**Figure S2**

**
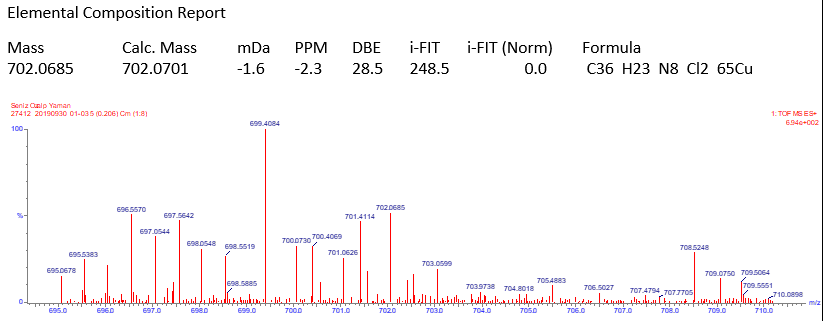
**

**Figure S3**

**
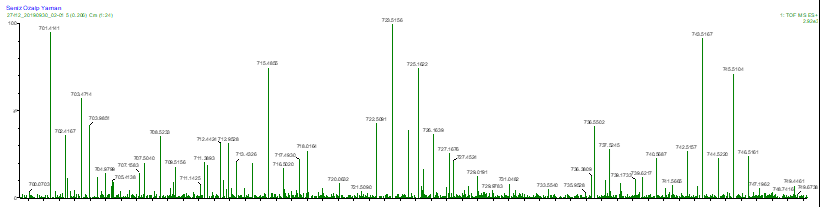
**

**Figure S4.**

**
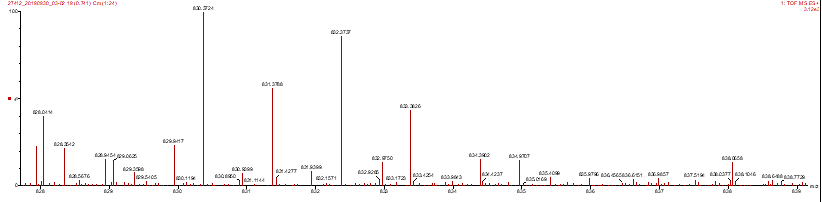
**

**Figure S5**

**
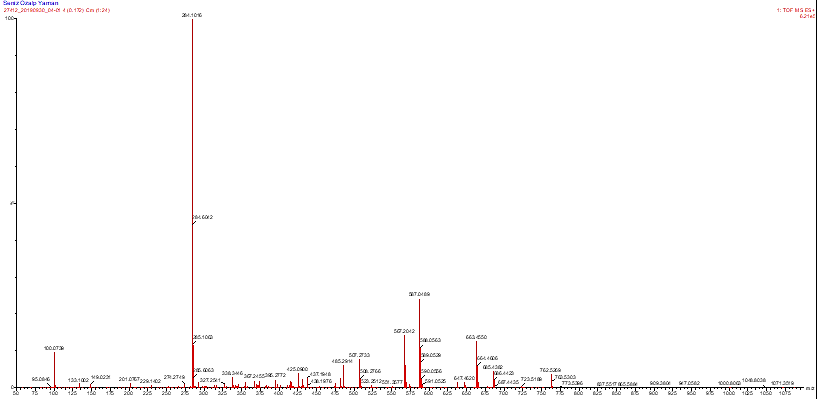
**

**Figure S6**

**Figure S7**

**Figure S8**

**Figure S9**

**Figure S10**

**
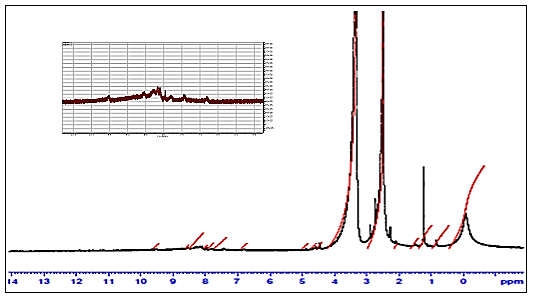
**

**Figure S11**

**
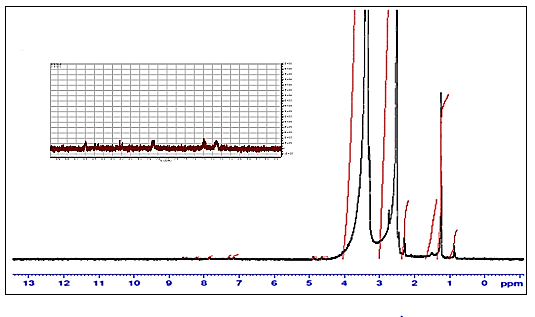
**

**Figure S13**

**
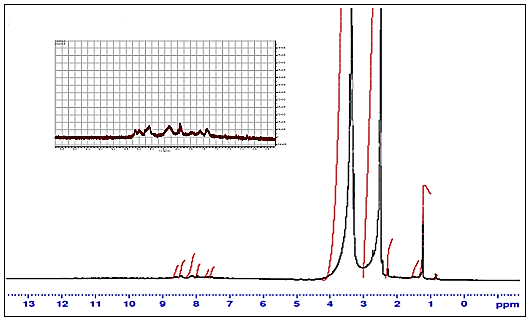
**

**Figure S12**

**
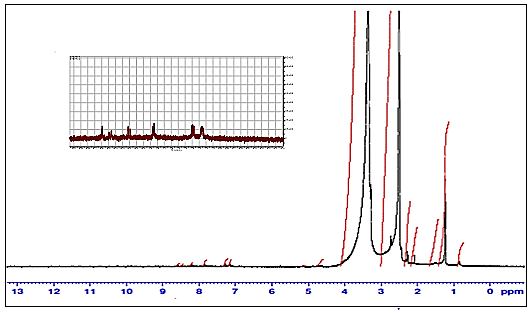
**

**Figure S14**

**_
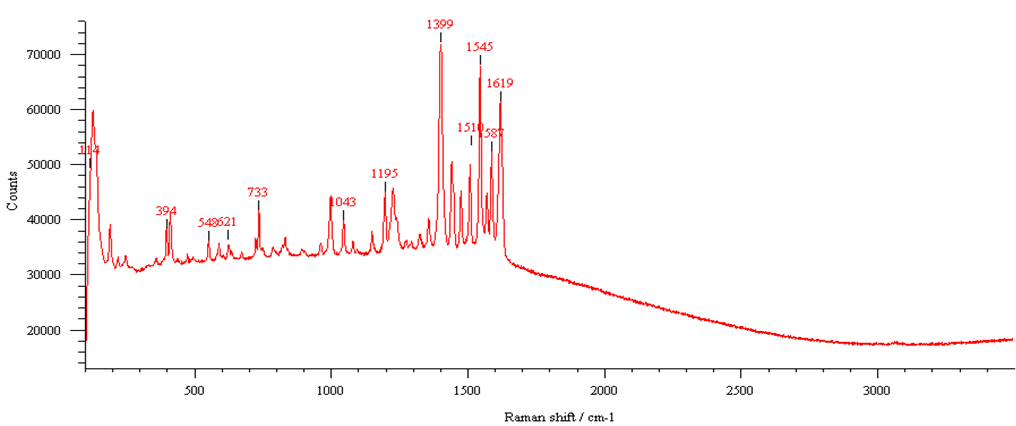
_**

Cu-N

Cu-Cl

**Figure S15**

**_
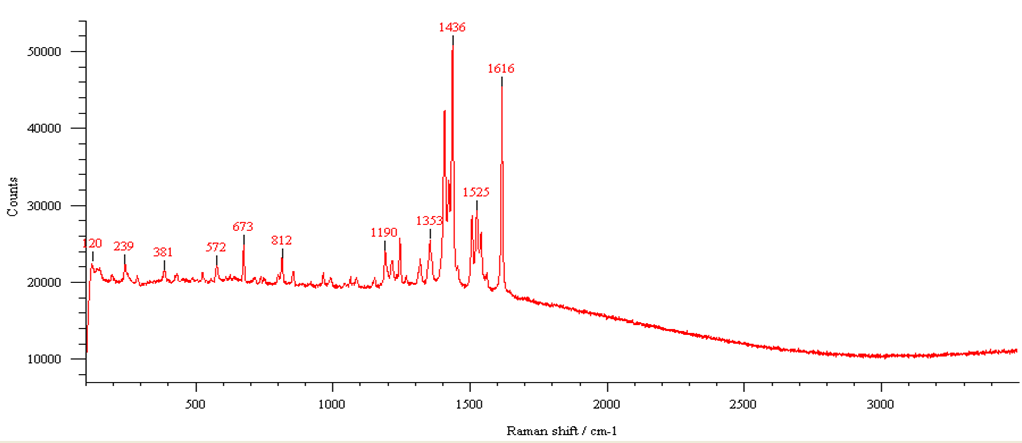
_**

Cu-N

Cu-Cl

**Figure S16**

**_
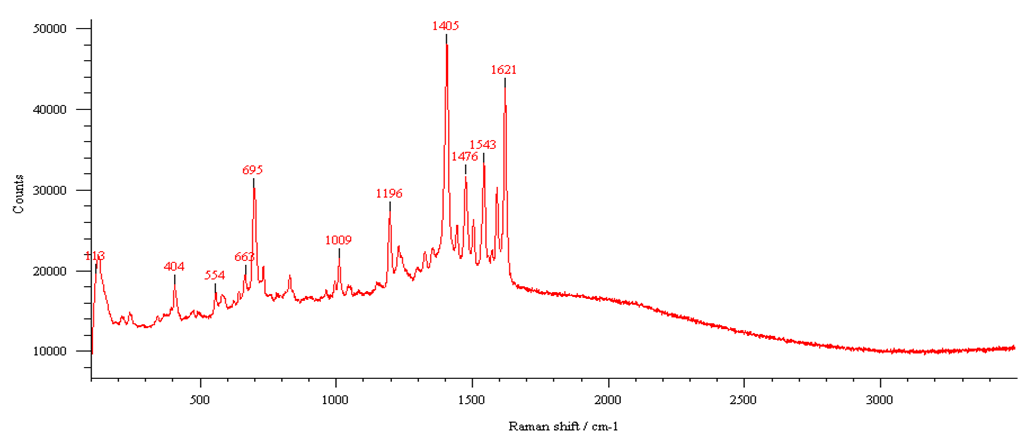
_**

Pt-N

Pt-Cl

**Figure S17**

**_
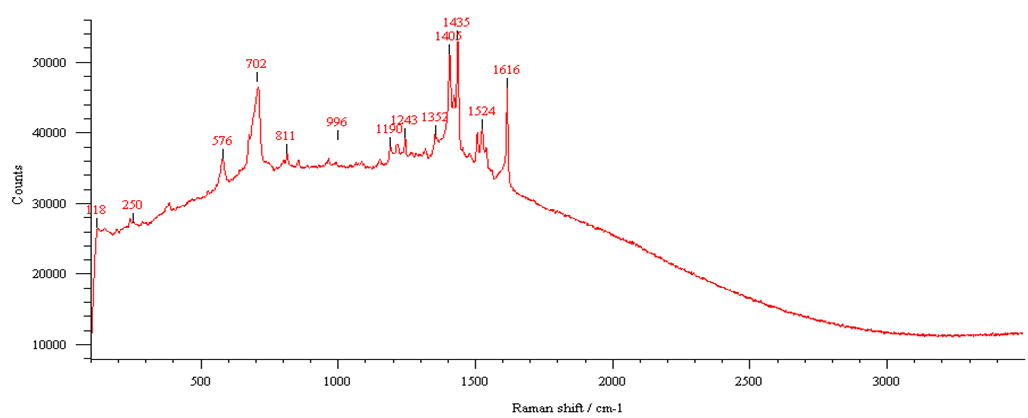
_**

Pt-N

Pt-Cl

**Figure S18**

**B**

**A**

**D**

**C**

**Figure S19**

**(b)**

**(a)**

**(c)**

**(d)**

**Figure S20**

**(a)**

**(b)**

**(d)**

**(c)**

**Figure S21**

**Figure S22**

**(b)**

**(a)**

**(d)**

**(c)**

**Figure S22**

**(b)**

**(a)**

**(d)**

**(c)**

**Figure S23**

**(a)**

**(b)**

**(d)**

**(c)**

**Figure S24**

**(a)**

**(b)**

**(d)**

**(c)**

**Figure S25**
